# Enhancing iron content and growth of cucumber seedlings with MgFe-LDHs under low-temperature stress

**DOI:** 10.1186/s12951-024-02545-x

**Published:** 2024-05-19

**Authors:** Hongyang Wu, Xiaoyang Wan, Jiefei Niu, Yidan Cao, Shufang Wang, Yu Zhang, Yayu Guo, Huimin Xu, Xian Xue, Jun Yao, Cuifang Zhu, Yang Li, Qiang Li, Tao Lu, Hongjun Yu, Weijie Jiang

**Affiliations:** 1 State Key Laboratory of Vegetable Biobreeding, Institute of Vegetables and Flowers, Chinese Academy of Agricultural Sciences, Beijing, 100081 China; 2 College of Horticulture, Xinjiang Agricultural University, Urumqi, 830052 China; 3 Research Unit of Molecular Epidemiology, Helmholtz Zentrum München, Neuherberg, 85764 Germany; 4 Faculty of Medicine, Ludwig- Maximilians-University München, Munich, 81377 Germany; 5 Institute of Botany, Chinese Academy of Sciences, Beijing, 100093 China; 6 Faculty of Environmental Science and Engineering, Kunming University of Science and Technology, Kunming, 650500 China; 7 College of Biological Sciences and Technology, Beijing Forestry University, Beijing, 100083 China; 8 College of Biological Sciences, China Agricultural University, Beijing, 100193 China; 9 College of Agriculture, Henan University of Science and Technology, Luoyang, 471000 China; 10 Guangdong Provincial Key Laboratory of Silviculture, Protection and Utilization, Guangdong Academy of Forestry, Guangzhou, 510520 China

**Keywords:** MgFe-layered double hydroxide nanoparticles, Seedling emergence rate, Nitrogen fixation, Fe fertilizer

## Abstract

The development of cost-effective and eco-friendly fertilizers is crucial for enhancing iron (Fe) uptake in crops and can help alleviate dietary Fe deficiencies, especially in populations with limited access to meat. This study focused on the application of MgFe-layered double hydroxide nanoparticles (MgFe-LDHs) as a potential solution. We successfully synthesized and characterized MgFe-LDHs and observed that 1–10 mg/L MgFe-LDHs improved cucumber seed germination and water uptake. Notably, the application of 10 mg/L MgFe-LDHs to roots significantly increased the seedling emergence rate and growth under low-temperature stress. The application of 10 mg/L MgFe-LDHs during sowing increased the root length, lateral root number, root fresh weight, aboveground fresh weight, and hypocotyl length under low-temperature stress. A comprehensive analysis integrating plant physiology, nutrition, and transcriptomics suggested that MgFe-LDHs improve cold tolerance by upregulating SA to stimulate *CsFAD3* expression, elevating GA_3_ levels for enhanced nitrogen metabolism and protein synthesis, and reducing levels of ABA and JA to support seedling emergence rate and growth, along with increasing the expression and activity of peroxidase genes. SEM and FTIR further confirmed the adsorption of MgFe-LDHs onto the root hairs in the mature zone of the root apex. Remarkably, MgFe-LDHs application led to a 46% increase (*p* < 0.05) in the Fe content within cucumber seedlings, a phenomenon not observed with comparable iron salt solutions, suggesting that the nanocrystalline nature of MgFe-LDHs enhances their absorption efficiency in plants. Additionally, MgFe-LDHs significantly increased the nitrogen (N) content of the seedlings by 12% (*p* < 0.05), promoting nitrogen fixation in the cucumber seedlings. These results pave the way for the development and use of LDH-based Fe fertilizers.

**Graphical Abstract:**

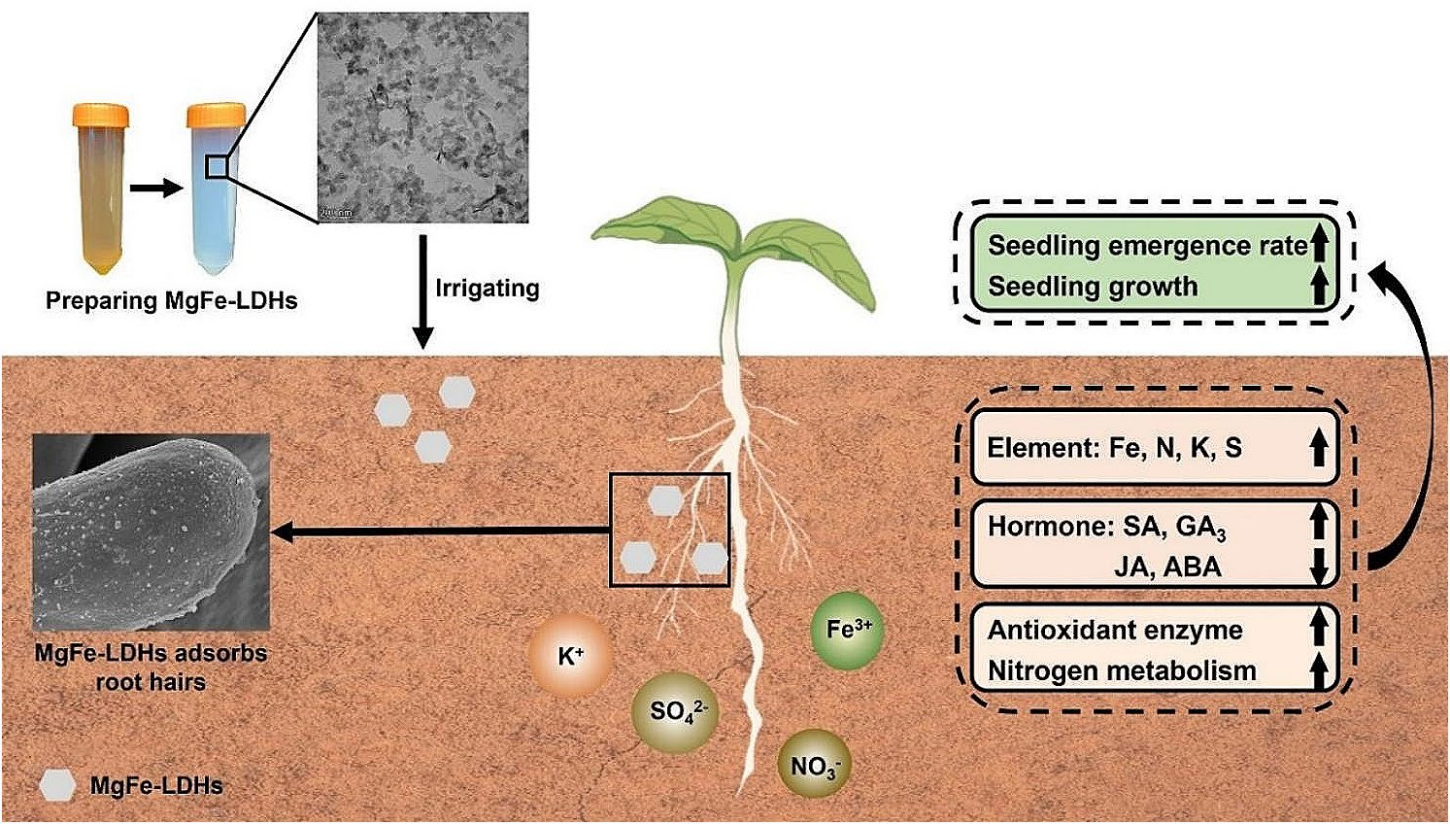

**Supplementary Information:**

The online version contains supplementary material available at 10.1186/s12951-024-02545-x.

## Introduction

Iron (Fe) is essential for human health, and its deficiency affects approximately 2 billion people, particularly infants, children, and pregnant women [1–3]. This can lead to iron deficiency anaemia, along with fatigue, dyspnoea, diminished physical performance, and cognitive deficits in both children and adults, while also increasing susceptibility to infections [4, 5]. The widespread Fe deficiency stems from the inability of economically disadvantaged countries or populations to obtain sufficient iron through meat consumption, coupled with inadequate iron from their staple crops, highlighting the urgency of enhancing crop Fe content [1–3]. Although Fe ranks as the fourth most plentiful element in Earth’s crust, its predominantly insoluble mineral state and restricted availability to plants contribute significantly to Fe deficiency in the majority of foods. To address Fe deficiency in agriculture, current practices frequently depend on the application of chelated iron, a strategy that is costly and potentially harmful to the environment [6]. According to Weinstein’s view, elevated concentrations of chelates can harm plants by competing with vital trace elements for binding sites on enzymes or proteins, particularly when the chelating agent’s affinity for metals exceeds that of cellular surface proteins [7]. Due to the critical role of Fe in agricultural production, significant interest has been directed towards developing innovative iron fertilizers, including iron lignosulfonate [8], CoFe_2_O_4_ nanoparticles [9], and Fe_2_O_3_ and Fe_3_O_4_ nanoparticles [10], in recent decades. For example, Zhang et al. demonstrated that nano-Fe can enhance the growth of alfalfa (*Medicago sativa* L.) by improving photosynthesis, nitrogen fixation, and antioxidant responses in nodules and altering the rhizosphere microbiome [11]. Similarly, Yu et al. reported that FeO NP fertilization could significantly increase rice (*Oryza sativa* L.) yield by regulating the activities of enzymes and enhancing nitrogen fixation, but they did not focus on the accumulation of iron in plants [12]. Additionally, the use of Fe_3_O_4_ nanoparticles coated with citrate (CA) or polyacrylic acid (PAA) to combat Fe deficiency in plants has been explored [13]. However, these fertilizers cannot replace chelated iron, and the development of green, efficient, and inexpensive new Fe fertilizers is still the key to solving the problem of plant trace element deficiency.

Layered double hydroxides (LDHs) have been widely used in the field of plant engineering [14, 15]. Many studies have been conducted on the use of small molecule drugs or dsRNA drugs for plant protection [16–18], some of which involve plant nanogene carriers [19–22]. Moreover, a few studies have reported that using LDHs at a proper concentration in a reasonable cultivation mode can promote crop growth, such as by enhancing the root elongation of Arabidopsis seedlings on 1/2 Murashige and Skoog media [15], and regular spraying of LDHs on the leaves can promote lettuce (*Lactuca sativa* L. var. ramosa Hort.) growth [23]. We do not, however, know whether LDHs can serve as nanofertilizers under real soil or substrate conditions, which highlights the need to study their potential effects on plant growth.

Cucumber (*Cucumis sativus*) is one of the main vegetable varieties consumed worldwide because of its economic and ecological relevance [24]. In this paper, we selected cucumber as a plant material to study the feasibility of using LDHs as a nanoiron fertilizer. As a continuation of our studies on LDHs in recent years [15, 23, 25], this study synthesized and characterized MgFe-LDHs and investigated their influence on cucumber seed germination, seedling emergence rate and growth under low-temperature stress and the associated cellular, physiological, and molecular mechanisms by combining transmission electron microscopy (TEM), scanning electron microscopy (SEM), Fourier transform infrared spectroscopy (FTIR), inductively coupled plasma‒optical emission spectrometry (ICP‒OES), and liquid chromatography‒mass spectrometry (LC‒MS). This study is the first to report that MgFe-LDHs can improve plant nutrition. Our overall aim was to investigate how the addition of MgFe-LDHs to a substrate affects cucumber root growth, which is useful for nanoiron fertilizer research using LDHs. We have successfully demonstrated that cucumber plants can absorb iron as MgFe-LDHs, an absorption that not only enhances their growth but also counteracts the detrimental effects of elevated iron levels, marking a significant stride in the advancement towards cultivating high-iron crops. By laying the groundwork for increasing the Fe content in the crops, our research paves the way for future advancements in functional food development. This could lead to significant health benefits, especially in regions where dietary Fe deficiency is prevalent. Overall, our study underscores the importance of nanotechnology in agricultural science and its potential to drive innovative solutions for global challenges in food security and nutrition.

## Experimental section

### Preparation and characterization of MgFe-LDHs

MgFe-LDHs were synthesized by a coprecipitation method. To prepare MgFe-LDHs, a solution containing 0.24 mol/L Mg(NO_3_)_2_·6H_2_O (99.99%; Macklin, China) and 0.06 mol/L Fe(NO_3_)_3_ (99.99%; Macklin, China) in 100 mL of deionized water (ddH_2_O) was sonicated and then stirred. Additionally, a 100 mL solution of 4 mol/L NaOH (99.99%; Macklin, China) was prepared separately. Under nitrogen protection and vigorous stirring at room temperature, both solutions were gradually added dropwise to a three-necked round-bottom flask containing 100 mL of ddH_2_O while maintaining the pH of the reaction mixture at 10 ± 0.2. The resulting viscous gel was then crystallized for 18 h in a completely sealed environment. Following crystallization, the product was centrifuged several times at 5,000 rpm for 8 min each and washed with water until the pH of the supernatant reached 7. Some of the obtained gel was dissolved in water to afford a 1 g/L solution. The size was measured using TEM (JEM-1230; Tokyo, Japan), and the zeta potentials of the MgFe-LDHs (1 g/L) were measured using a Malvern 2000 zeta potential analyser (Malvern Instruments, UK). The elemental contents of Mg and Fe in the MgFe-LDHs were quantitatively analysed by ICP‒OES (ICPOES730; Agilent, USA). The raw material consisted of Mg(NO_3_)_2_ and Fe(NO_3_)_3_ in quantities consistent with the amount of elemental Mg and Fe in the final product MgFe-LDHs. Some of the obtained gel was dried at 80 °C for 12 h for X-ray diffraction (XRD) analysis. Then, the patterns of the heated products were obtained using a Bruker D8 ADVANCE (Bruker, Germany) diffractometer equipped with Cu K*α* radiation operating at 40 kV and 40 mA. The patterns were recorded over a 2*θ* range from 5° to 80° with a scan speed of 5°/min. A Nicolet 6700 FTIR spectrometer (Nicolet, USA) was used to identify the functional groups in the nanoparticles across the wavenumber spectrum from 400 to 4,000 cm^− 1^ using a standard KBr pellet method with a sample-to-KBr ratio of 1:200.

### Seed germination, water uptake, seedling growth, and element content

Cucumber seeds (*Cucumis sativus* L. cv. Jinchun 4) obtained from the Tianjin Kernel Cucumber Research Institute were stored at 4 °C until use. This cultivar is resistant to downy mildew, powdery mildew, and Fusarium wilt and is suitable for spring/autumn open-field planting and late autumn greenhouse culture across China.

The germination experiment followed that of Wu [15], with minor adjustments. After sterilizing cucumber seeds in 0.1% KMnO_4_ (99%; Macklin, China) for 5 min and rinsing them three times with ddH_2_O, 40 seeds were placed on filter paper in a 90 mm Petri dish (Thermo Fisher Scientific, USA), with four dishes per treatment. Briefly, 10 mL of 0, 1, 10, or 100 mg/L MgFe-LDHs was added to the dishes under dark conditions at 25 °C. Germination rates were recorded every 24 h for five days.

The water uptake of seeds during imbibition was monitored using a protocol adapted from Bhardwaj et al. [26]. In each treatment group, 4 replicates were conducted, with 25 seeds per replicate. After weighing, the seeds were placed on filter paper (NewStar, China) in plastic dishes and incubated at 25 °C. To each dish, 10 mL of 0, 1, 10, or 100 mg/L MgFe-LDHs was added. At intervals of 3, 6, 12, 24, and 48 h, the seeds were removed, gently blotted to remove excess water, and reweighed. The increase in weight due to water absorption was calculated as a percentage of the seed’s dry weight using the following formula: WU% = 100 × (fresh weight of seed - dry weight of seed)/dry weight of seed, where WU represents water uptake.

The seedling growth experiment was carried out in the solar greenhouse and artificial climate chamber of the Institute of Vegetables and Flowers, Chinese Academy of Agricultural Sciences (CAAS), located in Beijing, China (116.33°E, 39.96°N). Cucumber seeds were disinfected with KMnO_4_, rinsed, and then incubated overnight in the dark on moist filter paper within Petri dishes at room temperature. The following day, seeds that had germinated, as evidenced by the emergence of a white radicle, were planted in a moist substrate (Pindstrup, Denmark) in 32-well seedling trays (Qingdao Lusheng, China) to a depth of 2 cm and then lightly covered. After planting, each seedling tray received 1 L of water (control check, CK for short), 10 mg/L MgFe-LDHs (LDH for short), or 10 mg/L raw material (RW for short). Each treatment consisted of a minimum of four seedling trays, with 32 seeds per tray. The outdoor temperatures are recorded in Additional file 1: Table S1, while the solar greenhouse temperatures are recorded in Additional file 1: Figure S2. A normal temperature simulation experiment was conducted in an artificial climate chamber at an average temperature of 25 °C, along with a low-temperature simulation experiment matching the mean daily temperatures outlined in Additional file 1: Figure S1. Ten days after the application of LDH and RW, growth parameters such as hypocotyl length, root length, lateral root number, aboveground fresh weight, and root fresh weight were measured. Seedling emergence rates were calculated by counting the number of emerged seeds and dividing by the total number of seeds sown. To ensure the accuracy of the results and minimize interference from other nutrients, the entire cultivation system was spared every 2–3 days to maintain substrate moisture.

The elemental contents of CK, LDH, and RW were quantified using ICP‒OES. After the 20 seedlings were phenotyped, they were divided into four groups, each containing both aboveground segments and roots. This arrangement resulted in four replicates per treatment, with each replicate comprising five seedlings. Then, the seedlings were rinsed five times with distilled water, dried at 65 °C, and ground into a fine powder. The concentrations of the following elements in lettuce were determined using ICP‒OES: iron (Fe), sulfur (S), magnesium (Mg), phosphorus (P), calcium (Ca), sodium (Na), and potassium (K). ICP‒OES elemental samples are recorded in Additional file 1: Table S2. An additional 20 seedlings were selected, and the aforementioned procedures were replicated to determine the nitrogen (N) content. The quantification of N was in accordance with methodologies detailed in previous research [27].

### SEM and TEM images

To examine the root surface structure, SEM was utilized. Cucumber roots were treated for 10 days in a greenhouse with CK, LDH, or RW (as detailed in “Seed germination, water uptake, seedling growth, and element content”) and subsequently dried and excised using a double-sided blade and tweezers. The samples were taken from the mature root zone located 4–5 cm from the root growth point. The specimens were then fixed in 2.5% glutaraldehyde for 4 h, followed by three washes in 0.1 M phosphate-buffered saline (PBS; pH 7.2). Dehydration was performed with a graded ethanol series (30–100%). After transfer to tert-butanol (Sigma, USA), the samples were lyophilized onto copper wire at 4 °C. Observations were conducted using an SU8020 scanning electron microscope (Hitachi, Japan).

To investigate the root structure, ultrathin sectioning and TEM were utilized. Samples were harvested from the mature root zone, which is located 3–4 cm from the root growth point. Then, they were carefully excised, washed with EDTA-2Na, fixed in glutaraldehyde, postfixed in osmium tetroxide, dehydrated in ethanol (30–100%), and embedded in Spurr’s resin (Sigma, USA), following the methods of Wu et al. [23, 28]. Subsequently, three roots per treatment were sectioned using an LKB-V ultramicrotome, stained with 2% uranyl acetate and 0.5% lead citrate, and viewed under a JEM-1230 transmission electron microscope (Tokyo, Japan) at 80 kV.

### Transcriptome sequencing

Ribonucleic acid (RNA) extraction and transcriptome profiling from cucumber roots followed the methods of Wu et al. [23]. Cucumber plants were treated for 10 days in a greenhouse with CK, LDH, or RW, as described in the “Seed germination, water uptake, seedling growth, and element content” section, with three replicates per treatment. For each replicate, three plants were randomly selected, and their roots were harvested as the experimental samples. To eliminate contaminants, the roots were washed with DEPC-treated water and then patted dry with filter paper. They were then excised using scissors and placed into 2 mL RNase-free cryogenic vials (Corning, China). After homogenization, the samples were placed in new RNase-free cryogenic vials (Corning, China) and snap-frozen in liquid nitrogen for 30 min. The samples were then stored at -80 °C. Sequencing was performed on an Illumina NovaSeq 6000 (Illumina, USA) using the TruSeq™ RNA Sample Preparation Kit. To identify differentially expressed genes (DEGs), genes/transcripts with a false discovery rate (FDR) < 0.05 and an absolute fold change (|FC|) ≥ 2 were considered significant. Additional details on the experimental workflow, read mapping, and data analysis are provided in Additional file 1: Section S1. The raw sequence data for the samples have been deposited in the NCBI database and are accessible at https://www.ncbi.nlm.nih.gov/sra/PRJNA1083769.

## Liquid chromatography‒mass spectrometry

To precisely quantify the contents of jasmonic acid (JA), salicylic acid (SA), abscisic acid (ABA), gibberellic acid (GA_3_), and indole-3-acetic acid (IAA), liquid chromatography–mass spectrometry (LC‒MS) was used. Cucumber plants were treated for 10 days in a greenhouse with CK, LDH, or RW as described in “Seed germination, water uptake, seedling growth, and element content”, with three biological replicates per treatment. After a 10-day growth period, roots were excised using a scalpel, rinsed with deionized water (ddH_2_O), and placed into a 2-mL Eppendorf (EP; Eppendorf, Germany) tube using tweezers. Approximately 20 cucumber roots were collected per EP tube to ensure a total sample mass of > 1 g. Subsequently, 1 mL of extraction solvent (methanol + 0.1% formic acid) was added to the sample, which was then ground into a homogenate. The volume was adjusted to 1 mL, and the tube was vortexed for more than 10 s, sonicated for 20 min, and then frozen at − 20 °C for at least 1 h. Next, the samples were centrifuged at 13,000×g for 5 min using a 5424R refrigerated centrifuge (Eppendorf, Germany). Then, 800 µL of the supernatant was removed, concentrated to dryness using a vacuum concentrator, and reconstituted in 100 µL of the initial mobile phase (10% acetonitrile–water with 0.1% formic acid). After vortexing with an MX-S vortex oscillator (Scilogex, USA), the sample was centrifuged at 13,000 rpm for 10 min. A total of 80 µL was pipetted into a glass liner and stored at − 20 °C for subsequent analysis. Analysis was conducted using an ACQUITY UPLC I-Class system (Waters, USA) coupled with a Xevo TQ-S micro triple quadrupole mass spectrometer (Waters, USA). Data acquisition was performed using MassLynx4.1 software (Waters, USA), and statistical significance was analysed using SPSS 18.0 software (IBM, USA).

### Real‑time quantitative analysis

The sampling method was consistent with that used for the transcriptome analysis. Root tissues were harvested and immediately flash-frozen in liquid nitrogen. Total RNA was extracted using an RN53 kit (Aidlab Biotechnologies, China) and reverse transcribed with One-Step SuperMix (TransGen Biotech, China). Quantitative real-time polymerase chain reaction (qRT‒PCR) was performed in triplicate for four biological replicates on an ABI PRISM 7300 system (Bio-Rad, USA) using SYBR Green Mix (TransGen Biotech, China) and 0.3 µM primer with 10 ng cDNA. The qRT‒PCR conditions included initial denaturation at 95 °C for 5 min, followed by 40 cycles of 95 °C for 20 s, 54 °C for 20 s, and 72 °C for 20 s, with a final extension at 84 °C for 30 s and elongation at 72 °C for 10 min. Melting curve analysis was subsequently performed. Relative mRNA expression levels across tissues were determined using the comparative Ct method as outlined in the iCycler manual (Bio-Rad, USA). Actin was used as an internal control due to its amplification efficiency similar to that of the target genes [29]. The sequences of the primers used for *CsFAD3* (*Csa1M532210*) and *CsFAD2.1* (*Csa3M808360*) were obtained from the literature [29]. The sequences of the *CsaV3_5G033660*, *CsaV3_6G002170*, *CsaV3_6G047430*, *CsaV3_7G003750*, *CsaV3_7G030380*, and *CsaV3_7G030390* primers are provided in Additional file 1: Table S3.

### Statistical analyses

The data were subjected to analysis of variance (ANOVA) for statistical analysis. Post hoc comparisons among mean values were conducted using the least significant difference (LSD) test at a significance threshold of *p* < 0.05, with SPSS 18.0 and Microsoft Excel 2023 employed for these analyses. Graphical representations were generated utilizing GraphPad Prism 9.5 and OriginPro 2022 software.

## Results and discussion

### Synthesis and characterization of MgFe-LDHs

**Fig. 1:**
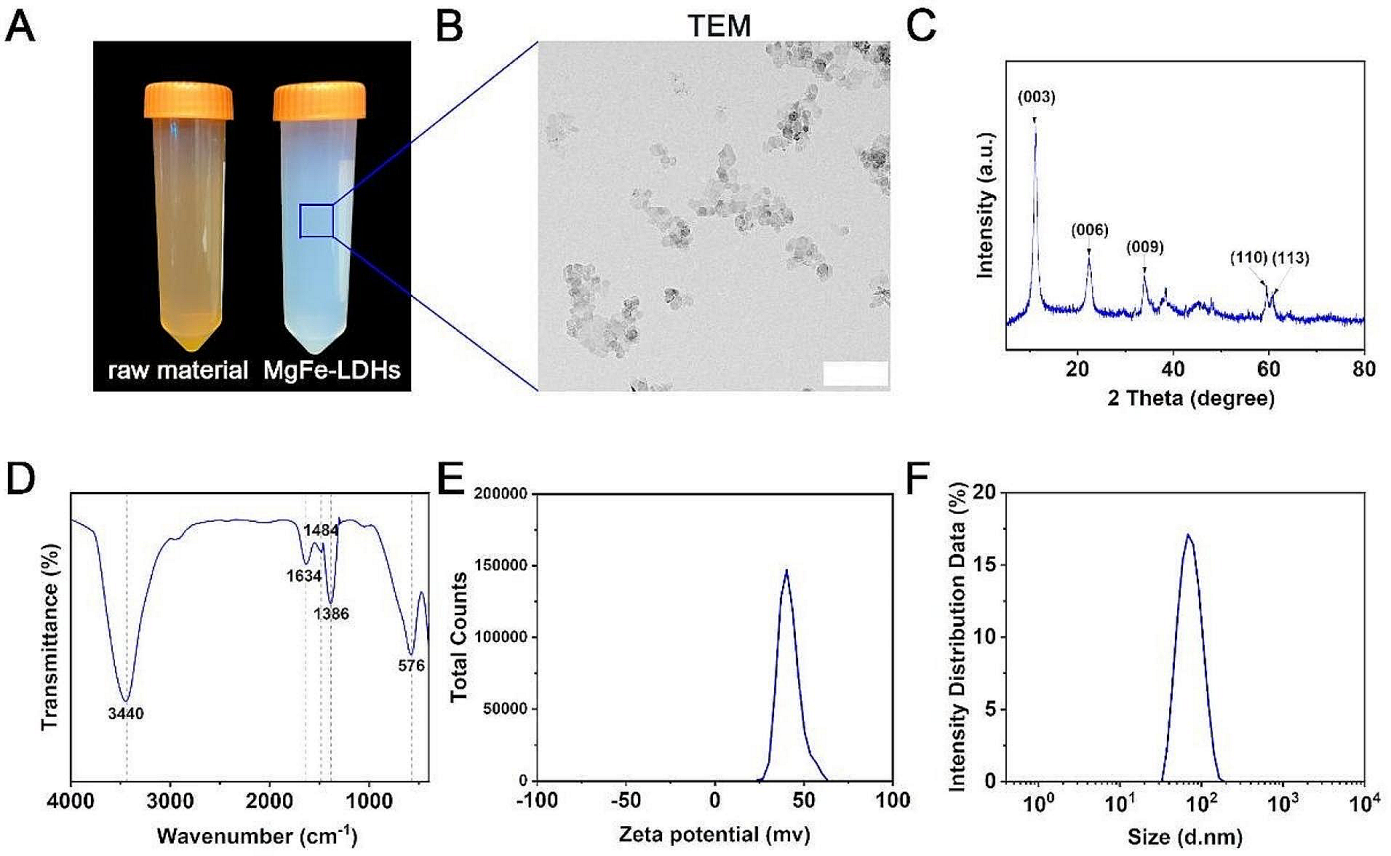
Synthesis and characterization of MgFe-LDHs. (**A**) Images of MgFe-LDHs and the raw material solutions. (**B**) TEM images of MgFe-LDHs; scale bar = 200 nm. (**C**) X-ray diffraction results of MgFe-LDHs. (**D**) FTIR spectroscopy of MgFe-LDHs. (**E**) Particle size distribution of MgFe-LDHs. (**F**) Mean zeta potential of MgFe-LDHs measured by a Nano Zetasizer

Brown Mg salts and Fe salts were used to synthesize colorless and transparent MgFe-LDHs by the coprecipitation method (Fig. 1A). The synthesized MgFe-LDHs showed excellent stability, with no signs of precipitation after storage at room temperature in a sealed container for three months. The crystal morphology and structure of the nanoparticles were characterized by TEM. The obtained TEM images revealed that the MgFe-LDH nanoparticles were hexagonal (Fig. 1B), which is consistent with previous reports [30]. In addition, XRD analysis of the chemical constitution and crystalline phase of the MgFe-LDHs (Fig. 1C) revealed distinct, symmetrical, sharp and well-defined diffraction peaks corresponding to the (003), (006), and (009) planes, indicating the successful preparation of nanomaterials with a complete layered structure, which is consistent with previous reports [30]. The measurement of the anion types as well as bonding types by FTIR spectroscopy, based on the vibrational patterns of the interacting infrared light and molecules, revealed differences in the intensities of the bands at 576, 1,386, 1,484, 1,634, and 3,440 cm^− 1^ (Fig. 1D). According to previous reports, the bands at 576 and 1,484 cm^− 1^ are attributed to Fe-O and Mg-O, respectively; the bands at 1,634 and 3,440 cm^− 1^ are attributed to the stretching of the O-H bond, mainly in the interlayer of the layered double hydroxide structure and water molecules adsorbed by them [31]. Consistent with a previous report of other MgFe-LDHs, the peak at 1,386 cm^− 1^ most likely corresponds to the vibrational absorption of interlayer NO_3_^−^ [30]. The results of zeta potential analyses (Fig. 1E) revealed that the average zeta potential of the MgFe-LDHs was + 41.6 mV, indicating that the MgFe-LDH particles were positively charged and that the dispersions were relatively stable. As shown in Fig. 1F, the particle size of the MgFe-LDHs was 69.47 nm, and the PDI was 0.075. Both the zeta potential and particle size distributions exhibited a normal distribution with a single peak, suggesting that the particles within the solution had similar size and charge characteristics and were evenly distributed and highly stable.

### MgFe-LDHs can promote cucumber seed germination, seedling emergence rate and seedling growth under low-temperature stress

**Fig. 2:**
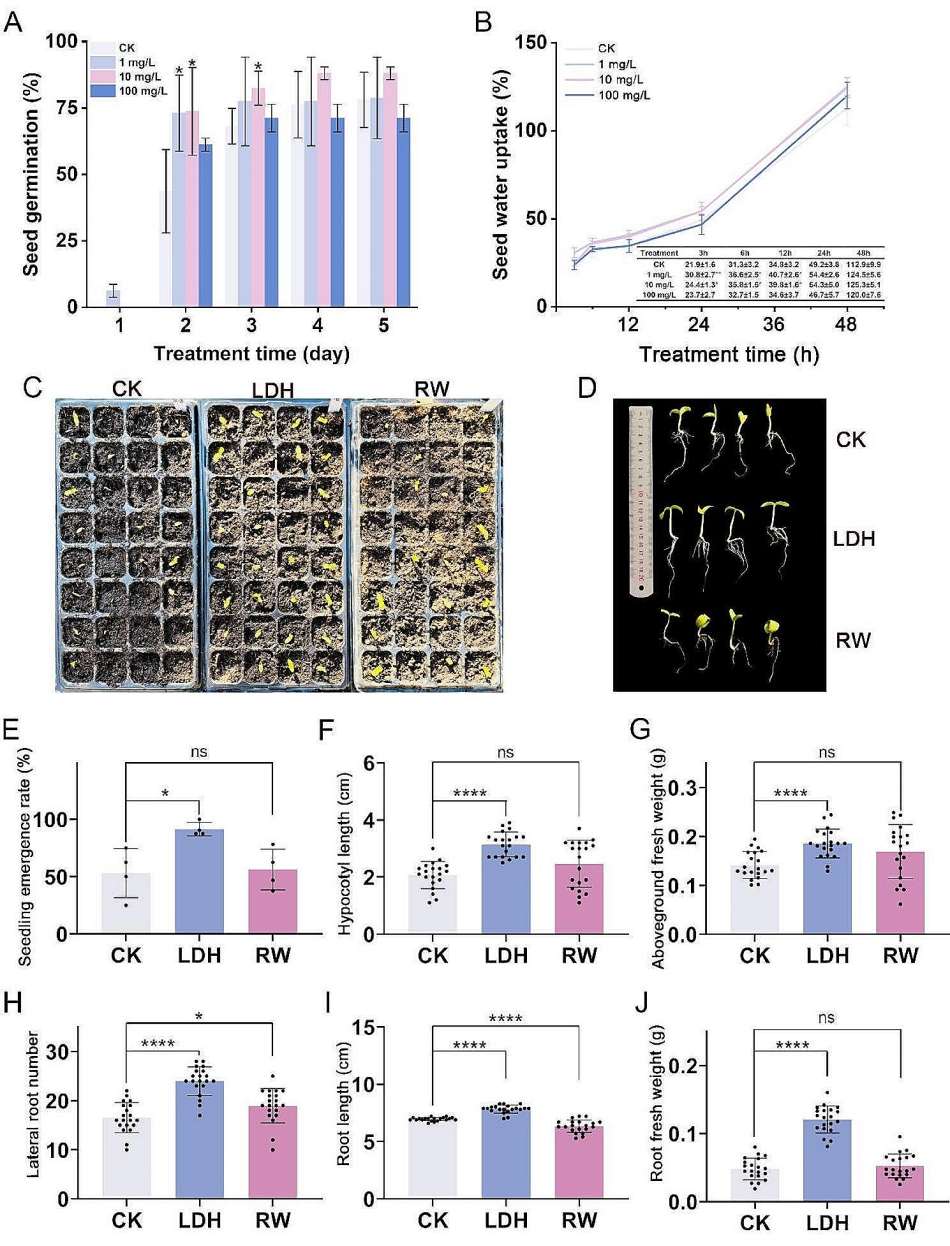
Effects of MgFe-LDHs on seed germination and seedling growth. Effects of 0, 1, 10, and 100 mg/L MgFe-LDHs on the seed germination (**A**) and seed water uptake (**B**) of cucumber plants. Cucumber seedling emergence rate (**C**) and phenotype (**D**) after 10 days of exposure to LDH and RW. The seedling emergence rate (**E**), hypocotyl length (**F**), aboveground fresh weight (**G**), lateral root number (**H**), root length (**I**) and root fresh weight (**J**) in cucumber roots following foliar exposure to LDH and RW after 10 days. The 10 mg/L MgFe-LDHs correspond to LDH. The 10 mg/L raw material corresponds to RW. *p < 0.05, **p < 0.01 and ****p < 0.0001, Student’s *t* test. The values are presented as the means ± SDs

Our results indicated that low nanomaterial concentrations (1–10 mg/L MgFe-LDHs) markedly increased the cucumber seed germination rate during the initial 3 days of seed germination. As shown in Fig. 2A, on day one, only 6.25 ± 2.5% of the seeds treated with 1 mg/L MgFe-LDHs germinated. However, by day two, the effect of treatment with either 1 mg/L (73.13 ± 14.34%) or 10 mg/L (73.75 ± 16.52%) on the germination rate was significantly greater than that of the CK (43.75 ± 15.66%, *p* < 0.05). On day three, the effect of the 10 mg/L (82.5 ± 6.45%) dose on the germination rate continued to significantly increase compared with that of the CK (68.13 ± 6.70%, *p* < 0.05). By day five, the cumulative germination rate was 78.13 ± 10.36% for the CK, 78.75 ± 15.34% for 1 mg/L, 88.13 ± 2.39% for 10 mg/L, and 71.25 ± 5.20% for 100 mg/L MgFe-LDHs. In addition, no statistically significant differences were detected on days four and five among all the MgFe-LDH treatments and the CK. Given that 1–10 mg/L MgFe-LDHs significantly increased seed germination during the first 3 days, with no subsequent effect, this beneficial effect appears to be specific to the early stages. Therefore, we measured seed water uptake in the early germination stage (the first 48 h), and the results (Fig. 2B) indicated that the initial imbibition phase was rapid, with seeds soaked in 1–10 mg/L MgFe-LDHs absorbing water significantly (*p* < 0.05) faster than those soaked in CK, particularly within the first 3 h (*p* < 0.01 at 1 mg/L; *p* < 0.05 at 10 mg/L). Indeed, nanomaterials have previously been reported to significantly increase seed water uptake in the rapid imbibition phase within 14 h, as has previously been observed with polystyrene nanoplastics (PSNPs) [32]. As the treatment time increased, no statistically significant differences in seed water uptake were observed after 12 h compared with those of the CK. Considering that both 1 and 10 mg/L of MgFe-LDHs increased seed water uptake and the germination rate during the early stages, as well as the final germination rate on day five for 10 mg/L compared with CK and 1 and 100 mg/L of MgFe-LDHs, it is reasonable to propose that the 10 mg/L concentration is the optimal dose for germination enhancement.

An investigation of the effect of 10 mg/L MgFe-LDHs (LDH) on cucumber seedling growth revealed a marked increase in the emergence rate on the 10th day of treatment (Fig. 2C), which prompted us to analyse the phenotype of the plants, and the resulting cucumber seedling morphology is shown in Fig. 2D. Compared to seedling emergence rate of 53.13 ± 21.3% of the CK group, LDH significantly (*p* < 0.05) increased the seedling emergence rate to 91.41 ± 5.9% (Fig. 2E). Conversely, treatment with 10 mg/L raw material (RW) did not affect the seedling emergence rate (56.25 ± 17.9%) (*p* > 0.05). As shown in Fig. 2F-J, we found that LDH significantly (*p* < 0.0001) increased the hypocotyl length by 51.7% (from 2.07 ± 0.47 to 3.14 ± 0.43 cm), aboveground fresh weight by 31.3% (from 0.141 ± 0.027 to 0.186 ± 0.030 g), lateral root number by 45% (from 16.55 ± 3.1 to 24 ± 2.9), root length by 12.5% (from 6.96 ± 0.15 to 7.83 ± 0.34 cm), and root fresh weight by 150.7% (from 0.048 ± 0.016 to 0.121 ± 0.020 g). In stark contrast, RW significantly inhibited (*p* < 0.0001) root length by 8.8% (from 6.96 ± 0.15 to 6.35 ± 0.55 cm) and significantly (*p* < 0.05) increased lateral root number (from 16.55 ± 3.10 to 18.95 ± 3.49). As shown in Additional file 1: Figure S1, monitoring the phenotypes after 20 days of exposure to LDH and RW revealed that LDH still significantly (*p* < 0.001) increased the root length by 39.1% (from 6.79 ± 1.12 to 9.45 ± 1.47 cm) and the lateral root number by 32.7% (from 20.5 ± 3.21 to 27.2 ± 3.29), indicating that the effect of LDH on seedlings is likely exerted on root growth and development in a sustained manner.

Cucumber, which originates from subtropical areas, is a chilling-sensitive plant with an ideal growth temperature range of 18–25 °C [33]. As early as 1865, Sachs reported that protoplasmic streaming in cucumber root hairs ceased at temperatures around 10–12℃ [34, 35]. Chilling-sensitive plant species exhibit a shared response to temperature, with the critical threshold for injury typically occurring between 10 and 12 °C [36]. The prevailing view is that low-temperature stress, including cold stress (0–20 °C) and freezing damage (< 0 °C), affects plants by changing cell membranes, reducing enzyme activities, and impairing nutrient uptake and metabolism, thus preventing normal growth [37]. In this study, we monitored Beijing’s outdoor temperatures (Additional file 1: Table S1) during an extreme cold event with temperature lows of -12 °C. The average of the temperatures (Additional file 1: Figure S2) in our greenhouse was 17.02 °C, but often dropped below 15 °C, reaching a low of 10.76 °C as outdoor temperatures decreased. Given that these temperatures were indeed within the low-temperature stress-inducing range (< 20 °C), it is reasonable to assume that the seedlings were subjected to low-temperature stress. The examination of root ultrastructure by TEM to further investigate the effects of low-temperature stress on seedling cellular structure revealed varying degrees of plasmolysis in the root systems of cucumber seedlings from the CK, LDH and RW treatment groups, showing deformed mitochondria with irregular shapes and indistinct cristae, severe twisting and deformation in some cells, and completely damaged organelle membrane structures, indicating chilling injury in the seedling roots (Additional file 1: Figure S3). Indeed, in plants, low-temperature stress causes increased electrolyte leakage from cells and plasmolysis, which are generally regarded as significant signs of chilling injury [38, 39].

The generalizability of MgFe-LDHs in enhancing seed germination and seedling growth was assessed by conducting simulation experiments in a controlled environment chamber at room temperature (Additional file 1: Figure S4). We found that LDH still significantly enhanced multiple aspects of seedling growth, including hypocotyl length by 19.5% (from 5.03 ± 0.63 to 6.01 ± 0.58 cm), aboveground fresh weight by 12.9% (from 0.418 ± 0.039 to 0.472 ± 0.037 g), root fresh weight by 29.4% (from 0.122 ± 0.021 to 0.159 ± 0.033 g) (*p* < 0.0001), and caused a significant increase of 16.1% in root length (from 7.21 ± 1.35 to 8.37 ± 1.45 cm) (*p* < 0.05), indicating the widespread enhancing effect of LDH on seedling growth. However, in the normal temperature simulation, it did not affect the seedling emergence rate (from 50 ± 17.7 to 62.5 ± 10.2%) (*p* > 0.05). In 2021, global cucumber production reached approximately 93,528,796 tons, with China accounting for 70% of the total production [37]. The northern regions of China face extreme climate challenges due to prolonged low and short-term critical low temperatures [37]. Considering the increasing occurrence of extreme climate events, increasing the seedling emergence rate and seedling growth at low temperatures holds practical significance for greenhouse production in northern China. Therefore, the focus of this study is not on normal temperature conditions but rather on low-temperature conditions. We conducted a low-temperature simulation experiment and obtained results consistent with the main findings in Fig. 2, showing the accuracy and generalizability of the results in Fig. 2. Compared with CK, LDH significantly improved the seedling emergence rate (from 77.34 ± 9.7 to 92.97 ± 8.2%; *p* < 0.05) and significantly increased root length by 34.8% (from 6.44 ± 1.49 to 8.68 ± 2.08 cm), root fresh weight by 63.7% (from 0.082 ± 0.017 to 0.135 ± 0.024 g), and lateral root number by 21.9% (from 17.52 ± 1.12 to 21.36 ± 2.53) (*p* < 0.0001) (Additional file 1: Figure S5). Based on the combined results shown in Fig. 2 and Additional file 1: Figures S4 and S5, we can confidently conclude that 10 mg/L MgFe-LDHs increased the cucumber seedling emergence rate and seedling growth under low-temperature stress. Therefore, we propose a new strategy to improve crop stress resistance by irrigating plants with 10 mg/L MgFe-LDHs during winter seedling cultivation in greenhouses, thereby improving resistance to low-temperature stress at the time of transplantation.

### MgFe-LDHs can be absorbed by cucumber plants and improve their nutritional status

**Fig. 3:**
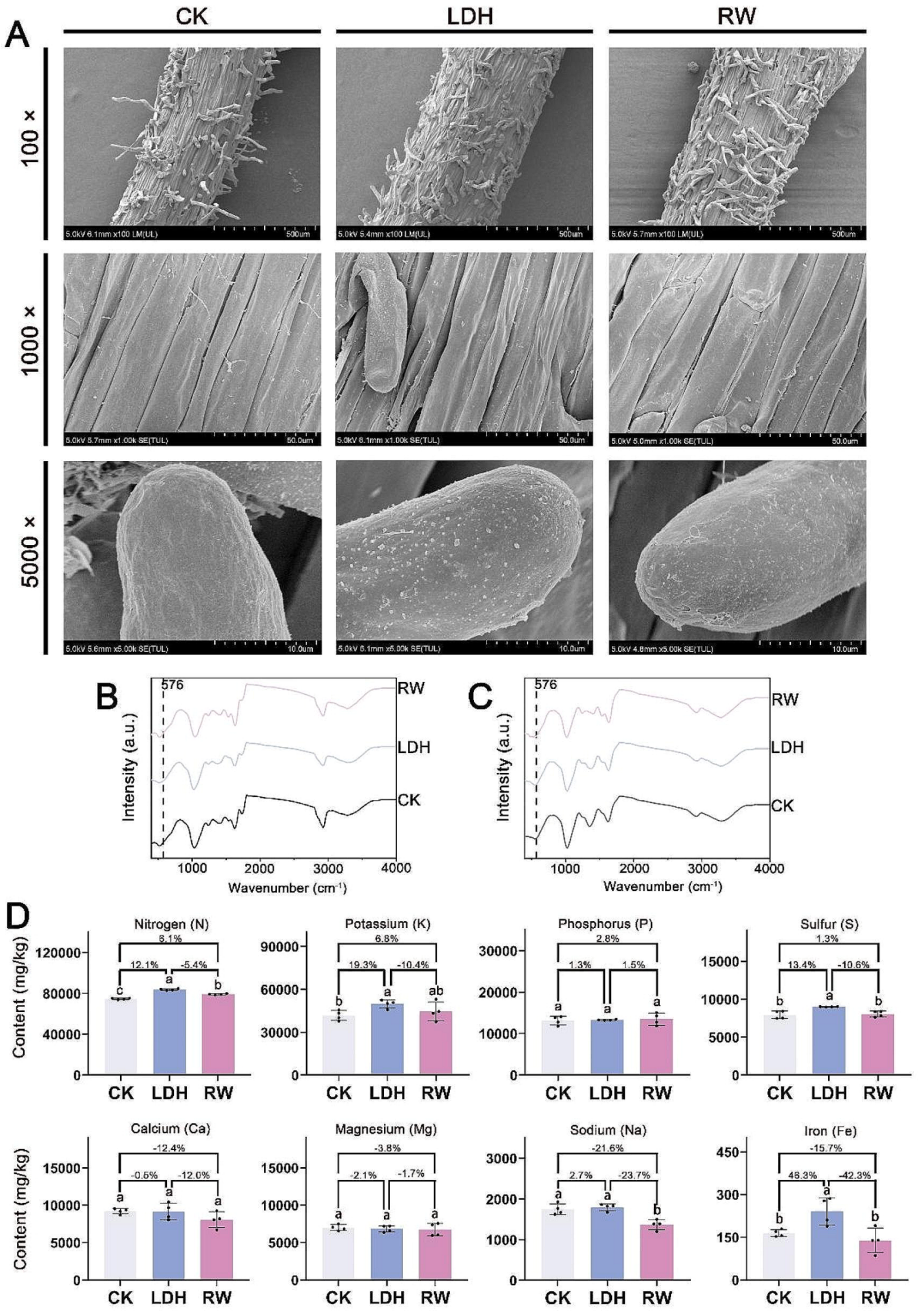
Cell fate of cucumber roots and nutrient element uptake by cucumber seedlings following 10 days of exposure to LDH and RW. (**A**) SEM images of cucumber seedling root tips. (**B**) FTIR spectrum of cucumber seedlings aboveground. (**C**) FTIR spectrum of cucumber seedling roots. (**D**) Element content in the dry matter of cucumber seedlings. Different lowercase letters indicate significant differences among treatments (*p* < 0.05). The percentages show the magnitude of change among the different treatment groups (LDH/CK, RW/CK, LDH/RW). The abbreviations used are the same as in Fig. 2

The ability of MgFe-LDHs to adsorb to and penetrate the cucumber root system was examined by obtaining SEM images of the mature zone at the root apex surface of plantlets from the CK, LDH, and RW treatment groups, as shown in Fig. 3A. SEM images at 100× magnification provided an overview of the morphology of the root maturation zone, while those at 1,000× magnification allowed detailed examination of the cell surface morphology within this zone. Furthermore, SEM images at 5,000× magnification revealed the surface morphology of the root hairs within the maturation zone. Morphological similarities were observed across the three treatment groups at both 100× and 1,000× magnification. However, SEM images at 5,000× magnification revealed a distinct surface morphology in the LDH treatment group compared to the RW and CK treatment groups. Notably, the mature region root hairs in the LDH group had numerous large flakes resembling those in Fig. 1B, contrasting with the fine particles, likely soil particulates, present on the root hair surfaces of the plantlets of the CK and RW groups. Given our previous finding that CuFe-LDHs can bind to the cell surface through hydrogen bonds [23], it is plausible that these flakes represent MgFe-LDHs. The preferential accumulation of MgFe-LDHs on root hair surfaces, rather than on the cell walls of the mature region, is probably due to the predominant absorption of water and nutrients by the plant through the root hairs in the mature zone. As a result, MgFe-LDHs co-accumulate on these surfaces alongside other nutrient elements.

Since FTIR spectroscopy can be used for qualitative analysis of LDHs [40], we used this technique to analyse the aboveground parts and roots of meticulously cleaned cucumber seedlings in order to validate the SEM results. The peaks in the range from 1,000 to 400 cm^− 1^ can be attributed to the lattice vibration modes of metal-oxygen (M-O) or metal-hydroxyl (M-OH) groups in the LDHs [40]. Therefore, in this study, we attributed the peak at 576 cm^− 1^ to MgFe-LDHs (Fig. 1D). We recorded the FTIR spectra of cucumber seedlings aboveground and roots, as shown in Fig. 3B and C, respectively. None of the three treatments of the cucumber seedlings aboveground showed a peak at 576 cm^− 1^, indicating that after 10 days of treatment, no MgFe-LDHs were present in the aboveground parts. However, the LDH-treated cucumber seedling roots showed a very weak Raman peak at 576 cm^− 1^ compared to that of the CK group, suggesting the possible presence of MgFe-LDHs in the interior and surface of the roots. Together, the results from SEM and FTIR spectroscopy analysis clearly indicate that MgFe-LDHs are capable of adsorbing onto the surface of root hairs. However, we were unable to determine whether MgFe-LDHs had directly entered the roots. Upon close examination of the TEM results (Additional file 1: Figure S3), MgFe-LDHs were not detected, possibly due to the thin composition of the MgFe-LDHs themselves. The thickness of the TEM sections, several tens of nanometers, makes it challenging to observe MgFe-LDHs in ultrathin sections. Thus, more sophisticated techniques are required to determine whether MgFe-LDHs enter root cells and to investigate their cellular fate within roots.

Considering that MgFe-LDHs are composed of metallic elements, we can indirectly determine whether MgFe-LDHs can be absorbed by plants through plant nutrient uptake. Compared to the CK group, LDH significantly increased the Fe content by 46.3% (from 164.5 ± 12.5 to 240.6 ± 48.5 mg/Kg), K content by 19.3% (from 41,735.5 ± 3,460.5 to 49,771.7 ± 2,853.5 mg/Kg), S content by 13.4% (from 7,934.2 ± 519.5 to 8,995.7 ± 39.8 mg/Kg), and N content by 12.1% (from 74,725 ± 763.2 to 83,800 ± 840.6 mg/Kg). In contrast to those in the CK group, most elemental changes in the RW group were not significant. However, it is noteworthy that the RW treatment increased the nitrogen content by 6.1% (from 74,725 ± 763.2 to 79,275 ± 543.9 mg/Kg) (Fig. 3D and Additional file 1: Table S4).

Plants can absorb Fe^2+^, Fe^3+^, or chelated iron from soil [41]. The majority of plants primarily take up Fe^2+^ or chelated iron, while members of the Poaceae family and a few other species are capable of absorbing Fe^3+^ [42]. Once inside the plant, Fe typically becomes immobilized in the Fe^3+^ form, which is less mobile [41]. In 1844, Cris demonstrated the greening of chlorotic grape leaves by applying iron salt solutions, and in 1860, Sachs and Molisoh confirmed that Fe is an essential micronutrient for plants [34]. The Fe content in plant dry matter typically ranges from 100 to 300 mg/kg, with < 50 mg/kg indicating deficiency, 50–250 mg/kg being optimal, and > 300 mg/kg considered excessive, although substantial variation occurs across different species, within various organs of the same plant, and across different parts of the same organ [1, 43–45]. In this study, no significant differences were found between the RW and CK groups (Fig. 3D), indicating that despite irrigation with Fe^3+^, it was not effectively absorbed, which is consistent with the literature [41]. Conversely, treatment with LDH significantly increased the Fe content by 46% (from 164.5 ± 12.3 to 240.6 ± 58.5 mg/kg) (*p* < 0.05), suggesting that cucumbers can directly utilize the Fe present in MgFe-LDHs.

Since cucumbers are potassium-dependent vegetables, they exert a significant influence on the root system of the plant, thus enhancing water and nutrient absorption [46]. Previous studies have identified a notable antagonistic relationship between Mg and K uptake, with increased potassium absorption inhibiting magnesium uptake [47]. In our study, the RW group exhibited negligible absorption of Mg and K. Remarkably, the LDH treatment group exhibited insignificant Mg absorption but caused a substantial increase in K absorption. Although LDH irrigation significantly increased Fe absorption by up to 46%, the Mg content, which is also present in MgFe-LDHs, remained unchanged. We hypothesize that upon entering root cells, MgFe-LDHs stimulate K absorption, subsequently facilitating the extrusion of Mg released from MgFe-LDH breakdown and maintaining a balanced Mg ion state. This hypothesis needs further experimental confirmation, such as ion flux measurements.

N is the most essential mineral nutrient for plants [48] and plays a critical role in the synthesis of amino acids, proteins, and nucleic acids [49]. Plant roots primarily take up nitrogen in the form of nitrate anions (NO_3_^−^) and ammonium cations (NH_4_^+^), with terrestrial plants favouring NO_3_^−^ [50]. As shown in Fig. 3D, our findings indicate that LDH treatment significantly increased (*p* < 0.05) the N content by 12.1% (from 74,725 ± 763 to 83,800 ± 841 mg/Kg), while compared with the CK treatment, the RW treatment also significantly (*p* < 0.05) increased the N content by 6.1% (from 74,725 ± 763 to 79,275 ± 544 mg/Kg). This may be attributed to the magnesium nitrate and iron nitrate present in RW and the NO_3_^−^ in LDH (Fig. 1). However, LDH had a more pronounced effect on promoting N absorption than did RW, resulting in a 5.7% increase over that of RW (from 79,275 ± 544 to 83,800 ± 841 mg/Kg). We hypothesize that MgFe-LDH enhances nitrogen metabolism and uptake, a hypothesis that we intend to validate in subsequent studies.

Plants depend on S as an essential nutrient, which they use in lipid synthesis and in regulating protein synthesis through the formation of disulfide bonds (-S-S-). Certain enzymes contain critical -SH groups at their active sites. S also serves as a ligand, binding with metal ions, such as Fe, Zn, and Cu, which is important for its integration into metalloproteins and enzymes. Plants primarily absorb S in the form of SO_4_^2−^ through their roots, a process that is coupled with N uptake [51]. In our study, LDH significantly increased the S content by 13.4% (*p* < 0.05), while RW led to a modest increase of 1.3% (*p* > 0.05). These findings are consistent with the N absorption data, where LDH increased N by 12% (*p* < 0.05) and RW by 6% (*p* > 0.05). Increased levels of Fe and S may enhance the activity of metalloproteins and increase specific enzyme activities, thereby promoting plant growth and development. For example, iron-sulfur (Fe-S) proteins are found in the plastids, mitochondria, cytosol and nucleus of plants, where they are essential for numerous physiological and developmental processes [52].

Fe deficiency not only impairs plant production but also ranks among the top ten health challenges in modern society, with a particularly high prevalence in women of childbearing age. It is the leading cause of anaemia, affecting at least 2 billion individuals worldwide [1]. Increasing the iron content directly in crops is of significant value, but iron predominantly occurs in nature as a component of compounds, making its direct utilization challenging [2]. Initially, iron supplements such as iron citrate, iron tartrate, or ferrous sulfate were used to meet plant Fe requirements, but iron ions often result in insoluble compounds, hindering plant absorption [53]. In the 1950s, it was first recognized that Fe could be added to hydroponic nutrient solutions in a chelated form with EDTA, an approach that continues to be used today [7, 54]. However, the high cost of chelated Fe makes it unsuitable for use in developing countries [2]. Additionally, high concentrations of chelated Fe can disrupt the distribution of trace elements, potentially competing with essential plant enzymes for these micronutrients [7]. Current biotechnological methods aim to enhance the ability of crops to utilize environmental iron through breeding and genetic engineering. However, these techniques are technically challenging and not broadly applicable across multiple species [2].

In this study, we determined that the zeta potential of MgFe-LDHs is + 41.6 mV (Fig. 1E), indicating that this material can persist in the natural environment for an extended period. MgFe-LDHs maintain stability for several months without sedimentation (Fig. 1A). A reported study also suggested that LDH nanomaterials exhibit good pH sensitivity, remaining stable under neutral and alkaline conditions while dissolving in acidic environments (pH < 4) [55]. This suggests that LDH nanomaterials could be suitable for use in alkaline soils and certain acidic soils on our planet. The results in Fig. 2 show that MgFe-LDHs can facilitate the germination of cucumber seeds and promote their growth, thus proving beneficial for plant development. Furthermore, the data in Fig. 3 reveal that irrigating cucumber seedlings with 10 mg/L MgFe-LDHs can increase the Fe content in the plant dry matter by 46%, significantly promoting the uptake of essential elements, such as N, K, and S, thereby improving plant nutrition. Based on these findings, we posit that MgFe-LDHs or other LDHs containing Fe(II) and Fe(III) have the potential to become a new generation of iron fertilizers, serving as a supplement to chelated iron. MgFe-LDHs present unique advantages in iron supplementation strategies compared to other traditional methods: (i) they boast broad applicability, potentially accommodating various soil types; (ii) unlike many other iron-containing nanomaterials, MgFe-LDHs exhibit the ability to selectively adsorb onto plant root hairs, a trait seldom reported for other iron nanomaterials [13]; and (iii) whereas increased iron content often inhibits growth in certain biological iron supplementation methods [2, 3], no such growth inhibition was observed in the present study. Moreover, due to their ease of synthesis and low cost, these materials could be widely used in developing countries.

LDHs, known for their excellent biocompatibility, have been extensively used as plant nanogene vectors in the field of plant sciences, including MgFe-LDHs [16–20, 25, 56]. This implies that a variety of LDHs can traverse the plant cell wall and cell membrane to be directly absorbed or adsorbed onto the cell wall, gradually releasing their metallic layers and interlayer anions. Combining previous research with our current findings, we propose a new strategy using LDHs-based nanogene vectors to directly increase the contents of Fe and other trace elements in crops by spraying or irrigation, with the aims of improving plant nutrition and addressing global micronutrient deficiencies, known as ‘hidden hunger’.

Our study has certain limitations, and there is a need to investigate the potential adverse effects of MgFe-LDHs, as well as their long-term effects on crops. Specifically, we propose the following areas for further exploration: (i) conducting long-term studies on cucumber plants to assess the impact of MgFe-LDHs on fruit yield and the nutritional composition of cucumber dry matter; (ii) selecting important food crops, such as rice, to determine whether there is any effect on the iron content within the grains; and (iii) in analysing our experiments, we found that cucumber plants can directly absorb Fe in MgFe-LDHs, but the direct addition of Fe^3+^ did not produce significant results. However, our study lacked a comparative analysis with chelated iron fertilizers, such as EDTA-Fe. We believe that these research avenues should be the primary focus of our future work. Furthermore, we encourage the scientific community to take an interest in these areas and contribute to the resolution of the aforementioned issues.

### MgFe-LDHs can induce physiological changes in cucumber seedling roots

**Fig. 4:**
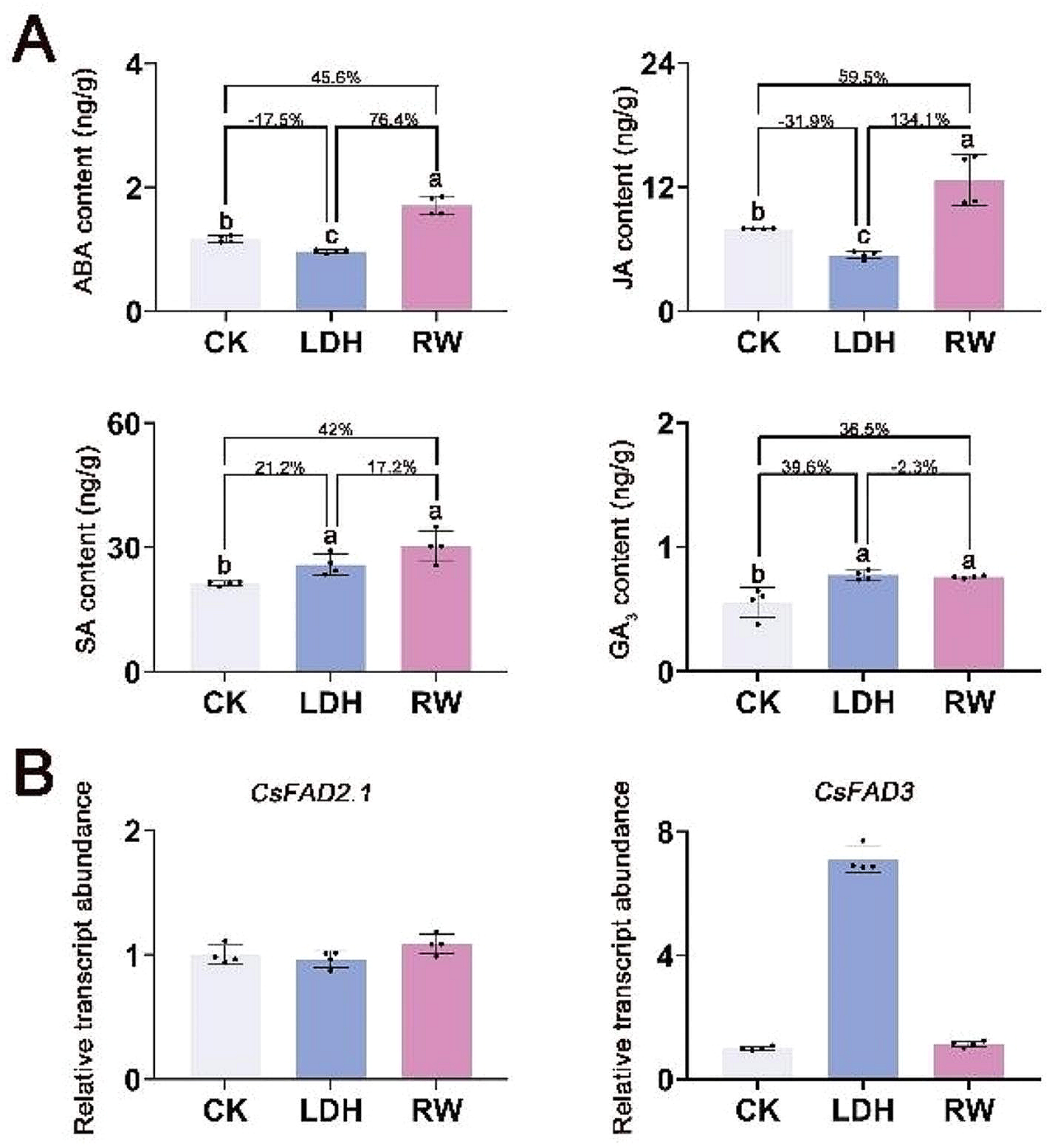
Root hormone content and *FAD* gene expression of cucumber roots following 10 days of exposure to LDH and RW. (**A**) Mass spectra of ABA, JA, SA, and GA_3_ contents. Different lowercase letters indicate significant differences among treatments (*p* < 0.05). The percentages show the magnitude of change among the different treatment groups (LDH/CK, RW/CK, LDH/RW). (**B**) LDH and RM influenced root *FAD* gene expression in cucumber seedlings. The abbreviations used are the same as in Fig. 2

Phytohormone biosynthesis and accumulation are essential for plant growth, development, and stress responses [57]. To determine whether LDH induces changes in plant hormones, we measured several hormones that have been reported to be associated with the response of cucumber plants to low-temperature stress, specifically ABA, JA, SA, GA_3_, and IAA [29, 58–60]. As shown in Fig. 4A, the application of LDH significantly (*p* < 0.05) increased the SA content by 21.2% (from 21.37 ± 0.54 to 25.90 ± 2.55 ng/g) and the GA_3_ content by 39.6% (from 0.56 ± 0.12 to 0.78 ± 0.04 ng/g), while it significantly (*p* < 0.05) decreased the levels of ABA by 17.5% (from 1.17 ± 0.06 to 0.97 ± 0.03 ng/g) and the JA content by 31.9% (from 7.97 ± 0.02 to 5.43 ± 0.36 ng/g). In contrast, the RW treatment led to a significant (*p* < 0.05) increase in SA by 42% (from 21.37 ± 0.54 to 30.34 ± 3.74 ng/g), GA_3_ by 36.5% (from 0.56 ± 0.12 to 0.76 ± 0.01 ng/g), ABA by 45.6% (from 1.17 ± 0.06 to 1.71 ± 0.20 ng/g), and JA by 59.5% (from 7.97 ± 0.02 to 12.71 ± 2.48 ng/g). These findings indicate that both the LDH and RW treatments increased the concentrations of SA and GA_3_ but had opposite effects on the levels of ABA and JA. Furthermore, the levels of the IAA hormone did not significantly differ among the three groups (Additional file 1: Figure S6), which is inconsistent with previous reports in which MgAl-LDHs were found to alter the IAA content [15], suggesting that LDHs with different elemental compositions elicit different hormonal responses in plant roots.

ABA plays a crucial role in seed development by regulating maturation and dormancy, as well as inhibiting germination and postgerminative growth to protect plants from adverse conditions [61, 62]. JA is recognized as one of the primary plant hormones that regulates stress responses by inducing defense mechanisms and the synthesis of specialized metabolites. Elevated levels of JA have often been correlated with reduced plant growth, presumably due to the redirection of carbon resources from primary growth processes to the biosynthesis of defensive compounds [63]. Recent research indicates that exogenous auxin acts synergistically with JA to enhance the ABA-induced delay of seed germination [62, 64]. In this study, RW simultaneously increased the contents of both ABA and JA (Fig. 4A), which explains the inhibition of root growth (Fig. 2I). Conversely, LDH simultaneously reduced the levels of ABA and JA, thus reducing the inhibitory effects of the two hormones. This may be one of the key mechanisms for promoting seedling growth and increasing the seedling emergence rate.

The exogenous application of SA, a pivotal signalling molecule that mediates plant responses to stress, has been shown to significantly enhance the cold tolerance of agricultural crops such as cucumber, tomato, and rice [65, 66]. Previous studies have shown that the exogenous application of salicylic acid during cold stress upregulates fatty acid desaturase (*FAD*) gene expression in cucumber seedling roots, increasing fatty acid unsaturation to enhance cell membrane stability and strengthen cold resistance [29, 67, 68]. The regulation of fatty acid unsaturation, catalyzed by FAD, which introduces double bonds into fatty acid chains to produce unsaturated fats, is critical to a plant’s ability to withstand chilling temperatures [68]. Arabidopsis mutants lacking FAD accumulate saturated fatty acids, leading to reduced chloroplast size and chlorophyll content, which impairs cold resistance, whereas the overexpression of FAD in tobacco increases unsaturated fatty acids, thus enhancing cold tolerance [69]. Comprehensive genomic analysis of cucumber revealed 23 *CsFAD* genes, most of which are upregulated in leaves in response to low-temperature stress [29]. In cucumber roots and hypocotyls, only *CsFAD2.1* and *CsFAD3* are significantly expressed, indicating their critical role in regulating fatty acid composition, unlike other FAD genes whose expression levels are limited [29]. Considering that among the 23 *CsFAD* genes, only two (*CsFAD2.1* and *CsFAD3*) showed significant expression, while the others showed minimal expression in both the roots and hypocotyls of cucumber seedlings, our study focused on these two critical *CsFAD* genes. Our findings revealed that *CsFAD2.1* expression remained relatively unchanged, whereas the expression of *CsFAD3* was increased up to 7-fold by LDH treatment (Fig. 4B). To our surprise, RW treatment increased the amount of SA hormone but did not upregulate the expression of *CsFAD3* (Fig. 4B). Although the precise mechanism leading to this phenomenon remains unknown, the most probable explanation is that the expression of *CsFAD3* is likely influenced by the uptake and utilization of nutrients such as N, P, and K through signalling pathways. Alternatively, Fe, as an essential cofactor for fatty acid desaturase (FAD), may be involved in the desaturation reaction by forming a complex with the enzyme [29, 41]. Our study revealed that treatment with LDH increased the Fe content in dry matter by 46% compared to that in the CK group (Fig. 3D), while treatment with RW did not significantly change the Fe content, suggesting a potential correlation between *CsFAD3* gene expression and Fe levels. Another possibility that cannot be excluded is that in the RW treatment group, other hormones regulate plant root growth by affecting the transcription of the *CsFAD3* gene. In any case, the finding that LDH treatment enhanced the SA hormone content and *CsFAD3* gene expression is beyond doubt. Given that there are many reports that the SA hormone can activate plant cold tolerance through the FAD enzyme [29, 67, 68], we conclude that LDH upregulates the expression of the *CsFAD3* gene by increasing the content of the SA hormone, thereby ultimately strengthening the plant’s resistance to cold.

GA, an essential hormonal regulator of plant growth, not only regulates the developmental trajectories of plants but also significantly contributes to their resilience against abiotic stresses, such as chilling temperatures, water deficit, and high salinity [70, 71]. Previous research has indicated that the application of GA_3_, either alone or in conjunction with other phytoregulators, enhances N uptake in agricultural crops [72]. Experimental data show that treatment with GA_3_ for 8 days increases cucumber seedling growth under suboptimal root-zone temperatures and nitrate (NO_3_^−^-N) absorption rates in the root system and leads to greater N accumulation in plant biomass [59]. Our findings suggest that both LDH and RW increase GA_3_ levels (Fig. 4A). The observed increase in N concentration in the plant dry matter for both the LDH and RW treatment groups (Fig. 3D) is consistent with previous studies [59, 72]. Thus, we can confidently assert that the increase in GA_3_ concentration induced by LDH facilitated increased N accumulation, contributing to the superior growth performance of LDH compared to that of CK. Additionally, we posit that LDH and RW may be involved in enhancing nitrogen metabolism; thus, it is imperative to substantiate this hypothesis through molecular-level studies, such as transcriptome analysis.

### MgFe-LDHs can induce transcriptomic changes in cucumber seedling roots

**Fig. 5:**
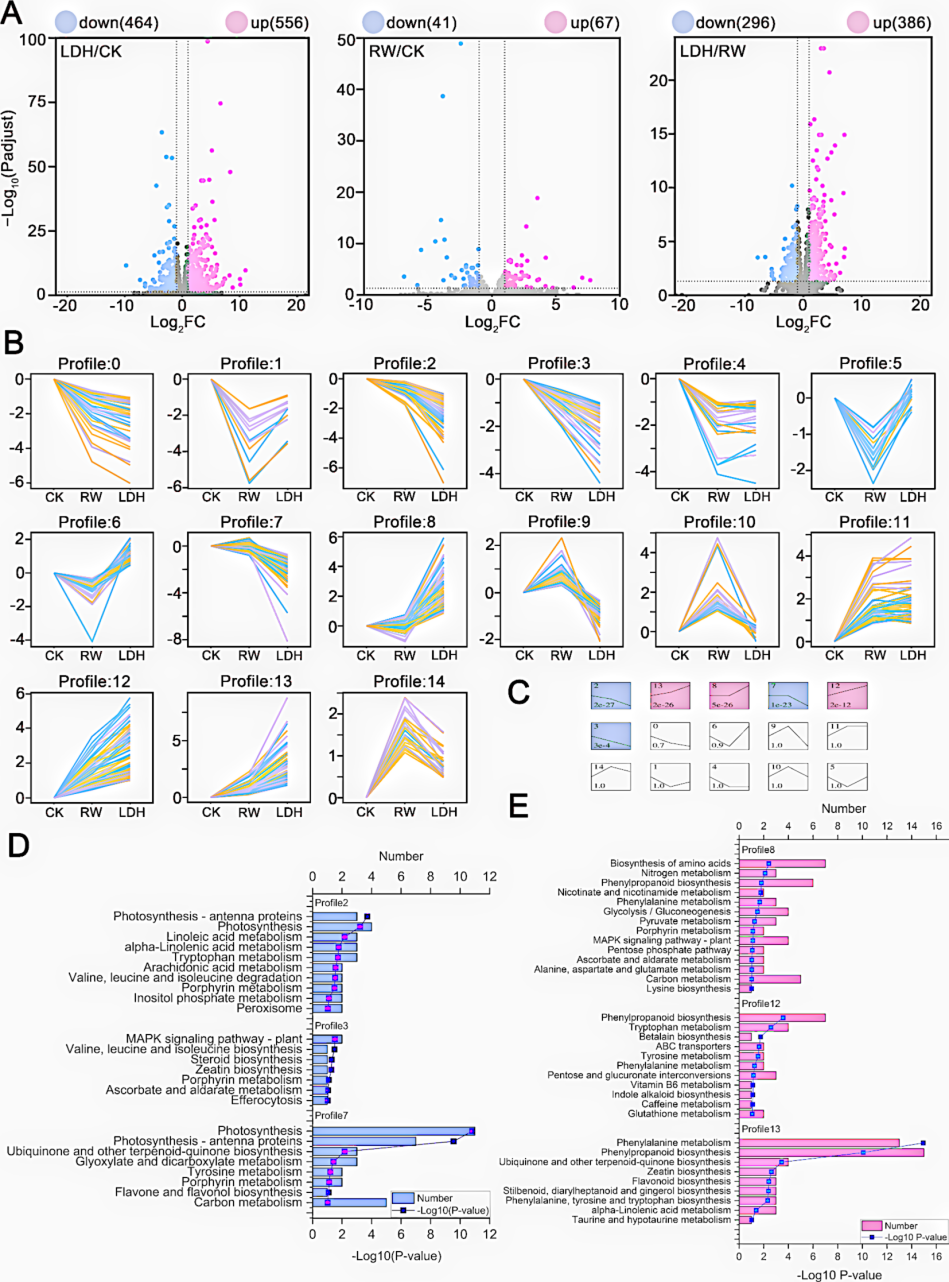
Transcriptome analysis of cucumber roots following 10 days of exposure to LDH and RW. (**A**) Volcano plots illustrating the DEGs with a false discovery rate (FDR) of less than 0.05 and an absolute fold change of ≥ 2 across various treatment groups (LDH/CK, RW/CK, LDH/RW). (**B**) Expression patterns of DEGs in response to LDH and RW treatments were deduced using short time-series expression miner (STEM) analysis. Each cluster represents the expression profile of all DEGs, which are depicted by colored lines. (**C**) Gene profiles are ranked based on the statistical significance (p value) of the number of genes assigned versus the expected count. (**D**) and (**E**) Kyoto Encyclopedia of Genes and Genomes (KEGG) pathway analysis identified significantly enriched profiles of differentially expressed genes in cucumber roots under LDH and RW treatments. The abbreviations used are consistent with those in Fig. 2

To elucidate the molecular mechanisms underlying cucumber seedling root resistance to low-temperature stress, we performed a transcriptomic analysis on roots treated with or without MgFe-LDHs. Our analysis of roots treated with LDH revealed a total of 1,020 DEGs, of which 556 were upregulated and 464 were downregulated compared to those in the CK group (Fig. 5A, Additional file 1: Figure S7). Moreover, in the roots of plants in the RW treatment group, there were 108 DEGs, of which 67 were upregulated and 41 were downregulated compared to those in the CK group (Fig. 5A, Additional file 1: Figure S7). In both the LDH and RW treatment groups, the number of upregulated DEGs was greater than the number of downregulated DEGs, with LDH inducing 9.4 times more DEGs than RW. Principal component analysis (PCA) of the transcriptomic data indicated that the LDH and CK treatment groups were clearly separated along component 1, which accounted for 31.3% of the total variance (Additional file 1: Figure S8). Considering that changes in gene expression represent one of several responses to environmental stress in plants [73], these results collectively suggest that LDH induces a broader spectrum of genetic changes than does CK. The comprehensive set of DEGs from the LDH and RW treatment groups were categorized into 15 distinct expression profiles (ranging from profile 0 to 14) using Short Time-series Expression Miner (STEM) software (Fig. 5B). Clustering analysis focused on the identification of significantly changed DEGs and revealed that profiles 2, 3, 7, 8, 12, and 13 exhibited the most significant (*p* < 0.01) changes (Fig. 5C). To deepen our understanding of the transcriptional changes, Kyoto Encyclopedia of Genes and Genomes (KEGG) pathway enrichment analysis was performed on genes in profiles 2, 3, 7, 8, 12, and 13 (Fig. 5D and E).

Among all profiles, profile 8 is notable for its representation of genes significantly affected by LDH in response to changes in RW and CK, without a reciprocal effect of RW on CK (Fig. 5B-E). Notably, profile 8 indicates that LDH enhances specific pathways, including biosynthesis of amino acids, nitrogen metabolism; nicotinate and nicotinamide metabolism; glycolysis/gluconeogenesis; pentose phosphate pathway; alanine, aspartate and glutamate metabolism; and lysine biosynthesis. These findings align with our findings demonstrating the role of LDH in increasing the N content (Fig. 3D), thereby promoting biosynthesis of amino acids, including lysine biosynthesis. The transamination of lysine with α-keto acids is crucial for the production of nonessential amino acids and enhances nitrogen cycling in organisms [74]. Furthermore, lysine biosynthesis stimulates the metabolism of alanine, aspartate, and glutamate, which are interconnected through the transamination of alanine with pyruvate, a core intermediate in glycolysis/gluconeogenesis [74, 75]. Additionally, NAD^+^ from nicotinate/nicotinamide metabolism and NADPH from the pentose phosphate pathway are essential cellular coenzymes involved in hydrogen transfer during biochemical reactions, contributing to material metabolism and energy conversion [74]. Since these coenzymes contain nicotinamide (C_6_H_6_N_2_O), they are implicated in nitrogen assimilation and metabolic processes.

Profiles 8, 12, and 13 revealed that both LDH and RW upregulated the phenylpropanoid biosynthesis and phenylalanine metabolism pathways, with LDH exhibiting a more pronounced effect (Fig. 5B-E). Phenylpropanoid biosynthesis and phenylalanine metabolism are critical to the comprehensive response of plants to both biotic and abiotic stresses [76]. These pathways serve as markers for plant stress responses, particularly when plants are exposed to variations in light conditions or mineral treatments. Furthermore, polymers derived from phenylpropanoids, such as lignin, suberine, and condensed tannins, significantly enhance the stability and resistance of gymnosperms and angiosperms against mechanical injuries and environmental stresses [76]. Lignin is one of the main components of the plant cell wall, enhancing its mechanical strength and improving plant tolerance to low temperatures [77]. Based on the above findings, we hypothesize that the upregulation of phenylpropanoid biosynthesis and phenylalanine metabolism by LDH could increase the content of lignin, thereby increasing the thickness of the cell walls in the mature root zone. To verify this hypothesis, we measured the thickness of the cell walls in Additional file 1: Figure S3, and as shown in Additional file 1: Figure S9, compared to that in the CK group, there was a slight increase in the thickness of the mature cell walls in the LDH treatment group (from 101.3 ± 34.3 to 125.4 ± 40.5 nm) (*p* < 0.01). The RW group also showed a slight increase relative to that of the CK group, but this increase was not as significant as that of the MgFe-LDH group (from 101.3 ± 34.3 to 112.3 ± 43.3 nm) (*p* > 0.05). Our results indicate that the upregulation of phenylpropanoid biosynthesis and phenylalanine metabolism by LDH may promote the accumulation of cell wall components, such as lignin, ultimately leading to a slight thickening of the mature cell walls, endowing cucumber seedlings with better stress resistance.

Profile 13 revealed that RW significantly enhances the gene pathways to CK, while LDH significantly enhances those to CK as well. Specific pathways include phenylalanine, tyrosine, and tryptophan biosynthesis; stilbenoid, diarylheptanoid, and gingerol biosynthesis; and taurine and hypotaurine metabolism (Fig. 5B-E). Hypotaurine serves as a precursor of taurine, both of which are sulfur-containing amino acids [78]. Phenylalanine, tyrosine, and tryptophan biosynthesis involves amino acid synthesis, while the synthesis of stilbenoid, diarylheptanoid, and gingerol originates from phenylalanine [74, 75]. LDH and RW induce these transcriptional pathways, and the most plausible explanation for this induction effect is that LDH and RW increase the content of N and S, with LDH inducing a greater increase than RW, and RW inducing a greater increase than CK. Therefore, LDH treatment improves plant nutrition, providing more nutritional elements for the synthesis of sulfur- and nonsulfur-containing amino acids, thereby promoting the expression of genes involved in relevant pathways. Furthermore, the order of the expression levels of these genes was LDH > RW > CK. In addition, within Profile 13, the Zeatin transcription pathway is involved in stress resistance across multiple species [79]. The ubiquinone and other terpenoid-quinone biosynthesis pathways mediate a variety of physiological processes, including plant cellular respiration, antioxidation, and signal transduction [80]. Alpha-linolenic acid metabolism can increase the content of unsaturated fatty acids to counteract the loss of cell membrane fluidity induced by adverse conditions [81]. Notably, in profiles 2, 3 and 7, the genes associated with these transcription pathways were significantly downregulated relative to those in the CK group, indicating complex physiological changes (Fig. 5B-D). Both LDH and RW upregulated and downregulated genes associated with these transcriptional pathways, requiring follow-up validation experiments.

Building on the STEM-KEGG findings, we independently performed Gene Ontology (GO) analysis for both the LDH and RW treatments, and the results are shown in Additional file 1: Figures S9, S10 and S11. The genes upregulated in response to LDH treatment were predominantly categorized under three major GO term categories: Molecular Function (MF), Cellular Component (CC), and Biological Process (BP). Notably, in the MF category, the most upregulated GO pathway was catalytic activity, which is associated with enzymes involved in biological processes [82]. This phenomenon can be plausibly attributed to the indispensable role of N as a constituent of proteins, including enzymes, while Fe, K, S, and other minerals act as cofactors or components of numerous enzymes [41]. The increase in the concentrations of Fe, N, K, S, and other nutritional elements caused by LDH could enhance enzyme synthesis, thereby influencing several key biochemical processes in plants, such as respiration, photosynthesis, and nitrogen fixation. Moreover, the increased concentrations of metal cations resulting from LDH treatment likely accounted for the upregulation in the MF category, specifically in pathways associated with iron ion binding, metal ion binding, and cation binding.

Remarkably, in the MF category, LDH upregulated the activity of xyloglucan: xyloglucosyl transferase, which is a key enzyme in the construction and strengthening of plant cell walls [83]. Additionally, the CC category revealed enrichment in genes involved in cell wall architecture. In the CC category, LDH upregulated pathways related to genes encoding components of the cell wall. In the BP category, LDH upregulated a multitude of pathways associated with cell wall biosynthesis and degradation, primarily including cell wall biogenesis, cell wall macromolecule metabolic process, cell wall polysaccharide metabolic process, and hemicellulose metabolic process. These observations, confirmed by the data in Fig. 5E and Additional file 1: Figure S9, preliminarily yet compellingly suggest that LDH treatment induces changes in the composition and structural integrity of root cell walls under low-temperature stress.

GO analysis revealed that LDH upregulated the response to stress. We identified 26 genes associated with stress responses (Additional file 1: Figure S13). Surprisingly, 15 of these genes were linked to peroxidases, 4 of which exhibited a log2-fold change (log_2_FC) greater than 6. High log_2_FC values indicate that these genes are highly expressed in the LDH group and significantly expressed at low levels in the CK group. By combining the volcano plot results (Fig. 5A), we found a total of 14 genes with a log_2_FC greater than 6, 4 of which were peroxidase genes. This led us to hypothesize that peroxidase genes might be the most significantly DEGs influenced by LDH. We randomly validated some of the peroxidase gene expression results by qRT‒PCR analysis, which were consistent with the transcriptome data, confirming the accuracy of the transcriptome analysis results (Additional file 1: Figure S14). Additionally, the measurement of peroxidase (POD) activity, as shown in Additional file 1: Figure S15, revealed that LDH significantly increased POD activity. Considering the role of Fe as the active site in peroxidases [41] and the involvement of N and S in protein and amino acid synthesis [74, 75], we propose that LDH likely facilitates the absorption of Fe, N, and S in plants. This, in turn, leads to the upregulation of the expression of genes involved in peroxidase synthesis, ultimately enhancing peroxidase synthesis and improving the cold stress resistance of the plant. The increase in peroxidase levels partly explains the decrease in ABA and JA hormone levels (Fig. 4), as well as the LDH-induced stress tolerance phenotype (Fig. 2). Alleviating excessive reactive oxygen species (ROS) levels is a powerful strategy for enhancing crop resilience, as oxidative bursts, characterized by rapid ROS release, are a major cause of damage in plants under stress [9]. Nanozymes, a class of nanomaterials endowed with intrinsic enzyme-mimetic activity capable of scavenging ROS, hold promise for application in agriculture, drawing on their extensive investigation in the biomedical field [9, 84]. For example, nano-CeO_2_, a well-known nanozyme, can improve plant growth under various abiotic stresses, including nitrogen deficiency, high salinity, heat, darkness and chilling [85]. Several other nanozymes, such as Fe_3_O_4_, MoS_2_, Mn_2_O_3_, and CoFe_2_O_4_, may have dual functionalities, potentially enhancing plant resilience to environmental stresses resulting from climate change [9]. In this study, MgFe-LDHs had similar effects, increasing the gene expression and activity of peroxidases and conferring resistance to low-temperature stress. Therefore, we hypothesize that MgFe-LDHs act as a type of nanozyme, analogous to the role of nanozymes such as Fe_3_O_4_.

The GO term enrichment analysis results for the LDH and RW treatments (Additional file 1: Figure S10 and S11) were consistent with the STEM-KEGG results (Fig. 5D and E). Notably, LDH downregulates cation binding and metal ion binding, which may be related to Mg absorption, while monoatomic ion transmembrane transporter activity and monoatomic cation transmembrane transporter activity may be associated with the promotion of K absorption. These findings may explain the results and our proposed hypothesis, as shown in Fig. 3.

In summary, using transcriptomic analyses, we elucidated and validated the results from previous chapters on cell biology, plant nutrition, and plant physiology. In addition, we elucidated the molecular mechanisms potentially associated with LDH-induced cold stress tolerance. It is clear that upon entering the plant, LDH increases the absorption of various nutrients, such as N, Fe, K, and S, while also influencing the levels of key plant hormones. By improving nutrient uptake and regulating hormone levels, LDH promotes the synthesis of key proteins, such as enzymes involved in nitrogen metabolism, ultimately leading to increased levels of peroxidases, thickening of the cell wall, and facilitating the growth of cucumber plants under cold stress conditions.

## Conclusions

The findings of our study significantly advance the understanding of the impacts of MgFe-LDHs on cucumber growth and nutrient absorption. A concentration of 10 mg/L MgFe-LDHs was found to optimally promote seed germination. When this concentration is applied during the transplantation of germinated cucumber seeds, it enhances seed emergence and seedling growth under low-temperature conditions, presenting a viable approach for improving greenhouse cucumber cultivation in harsh climates. Moreover, MgFe-LDHs were demonstrated to increase the uptake of essential nutrients such as N, K, and S, with a significant enhancement in Fe absorption. These findings suggest that LDH-based nanostructures could serve as direct nutrient supplies for nanofertilizer formulations. Our multidisciplinary research has provided insights into the cellular behavior of MgFe-LDHs and their role in enhancing resistance to stress and nutrient uptake in cucumbers. This comprehensive understanding lays the groundwork for the development of new nanofertilizers utilizing MgFe-LDHs.

### Electronic supplementary material

Below is the link to the electronic supplementary material.

Supplementary Material 1

## Data Availability

No datasets were generated or analysed during the current study.

## Abbreviations

ABA: Abscisic acid
BP: Biological process
Ca: Calcium
CC: Cellular component
CK: Control check
ddH_2_O: Deionized water
DEGs: Differentially expressed genes
FDR: False discovery rate
Fe: Iron
FTIR: Fourier transform infrared spectroscopy
GA_3_: Gibberellic acid
GO: Gene ontology
IAA: Indole-3-acetic acid
ICP‒OES: Inductively coupled plasma‒optical emission spectrometry
JA: Jasmonic acid
K: Potassium
KEGG: Kyoto encyclopedia of genes and genomes
LC‒MS: Liquid chromatography‒mass spectrometry
LDH: 10 mg/L MgFe-LDHs
LDHs: Layered double hydroxide nanomaterials
log_2_FC: log2-fold change
MF: Molecular function
Mg: Magnesium
N: Nitrogen
Na: Sodium
P: Phosphorus
PBS: Phosphate buffered saline
POD: Peroxidase
PSNPs: Polystyrene nanoplastics
qRT‒PCR: Quantitative real-time polymerase chain reaction
RNA: Ribonucleic acid
ROS: Reactive oxygen species
RW: 10 mg/L raw material
S: Sulfur
SA: Salicylic acid
SEM: Scanning electron microscopy
STEM: Short time-series expression miner
TEM: Transmission electron microscopy
XRD: X-ray diffraction
