# Supplementary material for: Enhancing iron content and growth of cucumber seedlings with MgFe-LDHs under low-temperature stress
Source: J Nanobiotechnology. 2024 May 19;22:268. doi: 10.1186/s12951-024-02545-x (PMC11103931; doi:10.1186/s12951-024-02545-x)
Supplement: Supplementary file 1 — Supplementary Material 1 [file 12951_2024_2545_MOESM1_ESM.docx]

Supporting information

**Enhancing iron content and growth of cucumber seedlings with MgFe-LDHs under low-temperature stress**

Hongyang Wu^1‡^*, Xiaoyang Wan^1‡^, Jiefei Niu^34‡^, Yidan Cao^1^, Shufang Wang^5^, Yu Zhang^6^, Yayu Guo^7^, Huimin Xu^8^, Xian Xue^9^, Jun Yao^10^, Cuifang Zhu^1^, Yang Li^1^, Hongjun Yu^1^*, and Weijie Jiang^12^*

*^1^ State Key Laboratory of Vegetable Biobreeding, Institute of Vegetables and Flowers, Chinese Academy of Agricultural Sciences, Beijing 100081, China.*

*^2^ College of Horticulture, Xinjiang Agricultural University, Urumqi 830052, China.*

*^3^ Research Unit of Molecular Epidemiology, Helmholtz Zentrum München, Neuherberg 85764, Germany.*

*^4^ Faculty of Medicine, Ludwig-Maximilians-University München, 81377 Munich, Germany.*

*^5^ Institute of Botany, Chinese Academy of Sciences, Beijing 100093, China.*

*^6^ Faculty of Environmental Science and Engineering, Kunming University of Science and Technology, Kunming 650500, China.*

*^7^ College of Biological Sciences and Technology, Beijing Forestry University, Beijing 100083, China.*

*^8^* *College of Biological Sciences, China Agricultural University, Beijing 100193, China.*

*^9^ College of Agriculture, Henan University of Science and Technology, Luoyang 471000, China.*

*^10^ Guangdong Provincial Key Laboratory of Silviculture, Protection and Utilization, Guangdong Academy of Forestry, Guangzhou 510520, China.*

**Keywords:** MgFe-layered double hydroxide nanoparticles, Cucumber seedlings, Low-temperature stress, Fe fertilizer

*Corresponding authors:  *Weijie Jiang*, *Hongjun Yu*, *Hongyang Wu*

State Key Laboratory of Vegetable Biobreeding, Institute of Vegetables and Flowers, Chinese Academy of Agricultural Sciences, Beijing 100081, P. R. China.

E-mail: [jiangweijie@caas.cn](mailto:jiangweijie@caas.cn); [yuhongjun@caas.cn](mailto:yuhongjun@caas.cn); [wuhongyang0829@foxmail.com](mailto:wuhongyang0829@foxmail.com)

^‡^ These authors contributed equally to this work.

**Additional file 1:**

**Section S1. Transcriptome analysis of cucumber roots**

**S1.1.** **The experimental workflow**

The experimental procedure entailed a multistep workflow: extraction of total RNA, enrichment of mRNA using Oligo dT beads, fragmentation of mRNA, synthesis of cDNA via reverse transcription, ligation of adapters, and sequencing on the Illumina platform. RNA was extracted from the tissue samples with a Nanodrop 2000 (Thermo Fisher Scientific, USA) to measure the RNA concentration and purity. RNA integrity was evaluated through agarose gel electrophoresis, and the RNA integrity number (RIN) was determined using an Agilent 2100 system (Agilent Technologies, USA). Each library preparation required a minimum of 1 μg of total RNA at a concentration of no less than 35 ng/μL, with an OD260/280 ratio of ≥1.8 and an OD260/230 ratio of ≥1.0. In eukaryotes, mRNA molecules are characterized by a poly-A tail at their 3' end. To selectively isolate mRNA from total RNA for transcriptomic analysis, magnetic beads coated with Oligo(dT) were utilized due to their specific affinity for the poly-A tail. The Illumina NovaSeq 6000 platform (Illumina, USA) was chosen for sequencing short sequence fragments. The isolated mRNA, consisting of full-length RNA sequences averaging several kilobases, needed to be fragmented. This was achieved by adding fragmentation buffer that induced random mRNA fragmentation. Magnetic bead selection was then used to isolate small fragments of approximately 300 bp in size. During reverse transcription, a random hexamer primer was added to initiate the synthesis of first-strand cDNA using the mRNA template. This was followed by second-strand synthesis, resulting in stable double-stranded cDNA. PCR amplification was carried out for 15 cycles. After amplification, the libraries were subjected to size selection using a 2% agarose gel to isolate 300 bp cDNA fragments. Library quantification was performed using the TBS380 assay (Invitrogen, USA). Bridge PCR amplification was conducted on the cBot instrument to generate clusters. Paired-end RNA sequencing was performed on a sequencer with a read length of 2 × 150 bp.

**S1.2.** **Read mapping**

**Table S5** presents the read counts, guanine content (GC%), guanine-cytosine content (GC%), and mean quality scores for the sequenced data. The precision and high quality of these datasets, coupled with efficient mapping to the cucumber genome and elevated rates of unique alignments, are deemed adequate for subsequent analytical endeavors. To corroborate the RNA-Seq results, six genes exhibiting differential expression were arbitrarily chosen, and their transcript levels were quantified using real-time quantitative PCR (qPCR). The log2-fold change (log_2_FC) and the direction of expression changes observed in the qPCR assays were in line with those from the RNA-Seq analysis. This concurrence substantiates the reliability of the RNA sequencing results and their appropriateness for further downstream analysis.

**S1.3. Differential expression analysis and functional enrichment**

The expression level of each gene was determined using the transcript count method to identify differentially expressed genes (DEGs) between two samples. RSEM (<http://deweylab.biostat.wisc.edu/rsem/>) was used to quantify gene abundances. Differential expression analysis was performed using DESeq2, with a p-adjusted value ≤ 0.05 and |log_2_FC|≥1 as the criteria for significance. Additionally, ClusterProfiler (<https://www.bioconductor.org/packages/release/bioc/html/clusterProfiler.html>) was utilized to conduct Kyoto Encyclopedia of Genes and Genomes (KEGG) pathway analysis.

## Figure

**
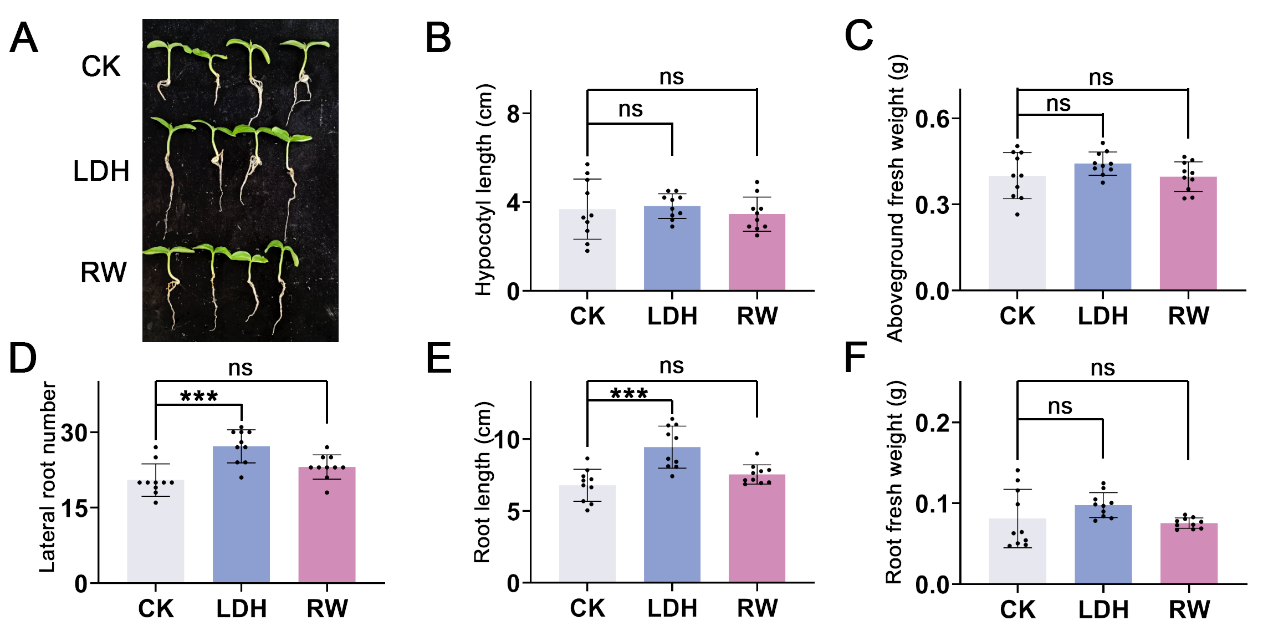
**

**Figure S1** Effects of MgFe-LDHs on plant growth after 20 days

Cucumber phenotype (**A**) after 20 days of exposure to LDH and RW; bar = 10 cm. The hypocotyl length (**B**), aboveground fresh weight (**C**), lateral root number (**D**), root length (**E**) and root fresh weight (**F**) of cucumber roots following foliar exposure to LDH and RW after 10 days. MgFe-LDHs = 10 mg/L are LDH for short. RW = 10 mg/L are RW for short. ****p* < 0.001, Student’s *t* test. The values are presented as the means ± SDs.


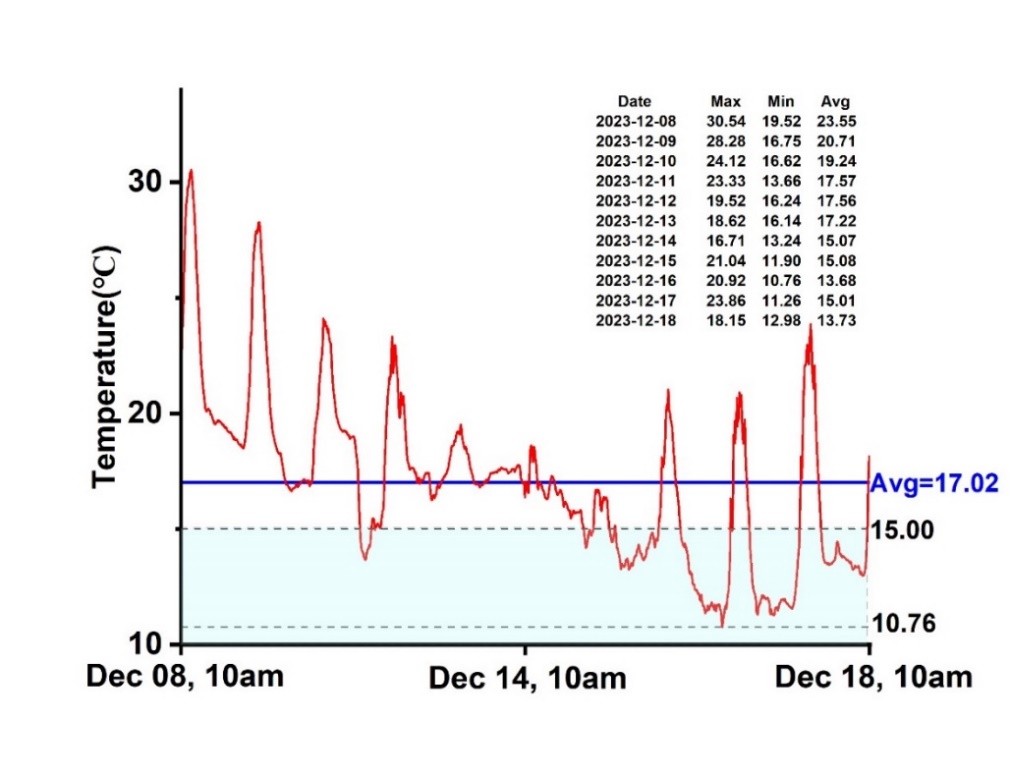


**Figure S2** Greenhouse temperature

**
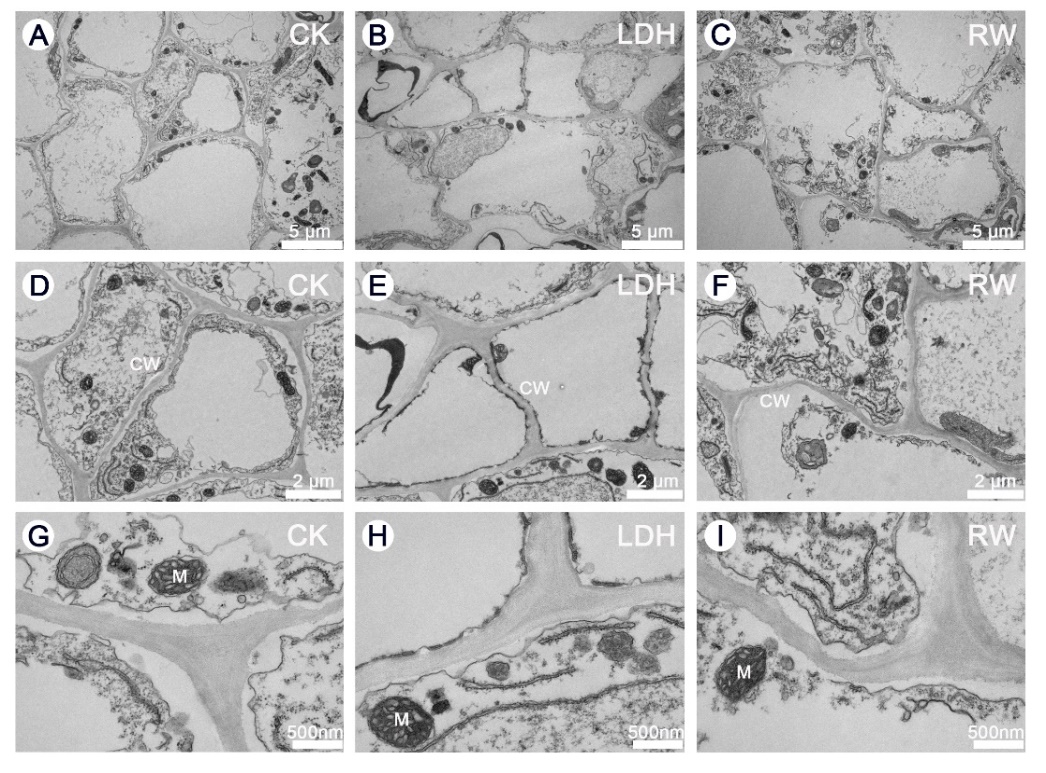
**

**Figure S3** The ultrastructure of the root tip cells of cucumber seedlings

(**A**, **D**, **G**) CK, (**B**, **E**, **H**) LDH, (**C**, **F**, I) RW. CK, Control check. CW, cell wall. M, Mitochondria. The abbreviations used are the same as those in Figure S1.

**
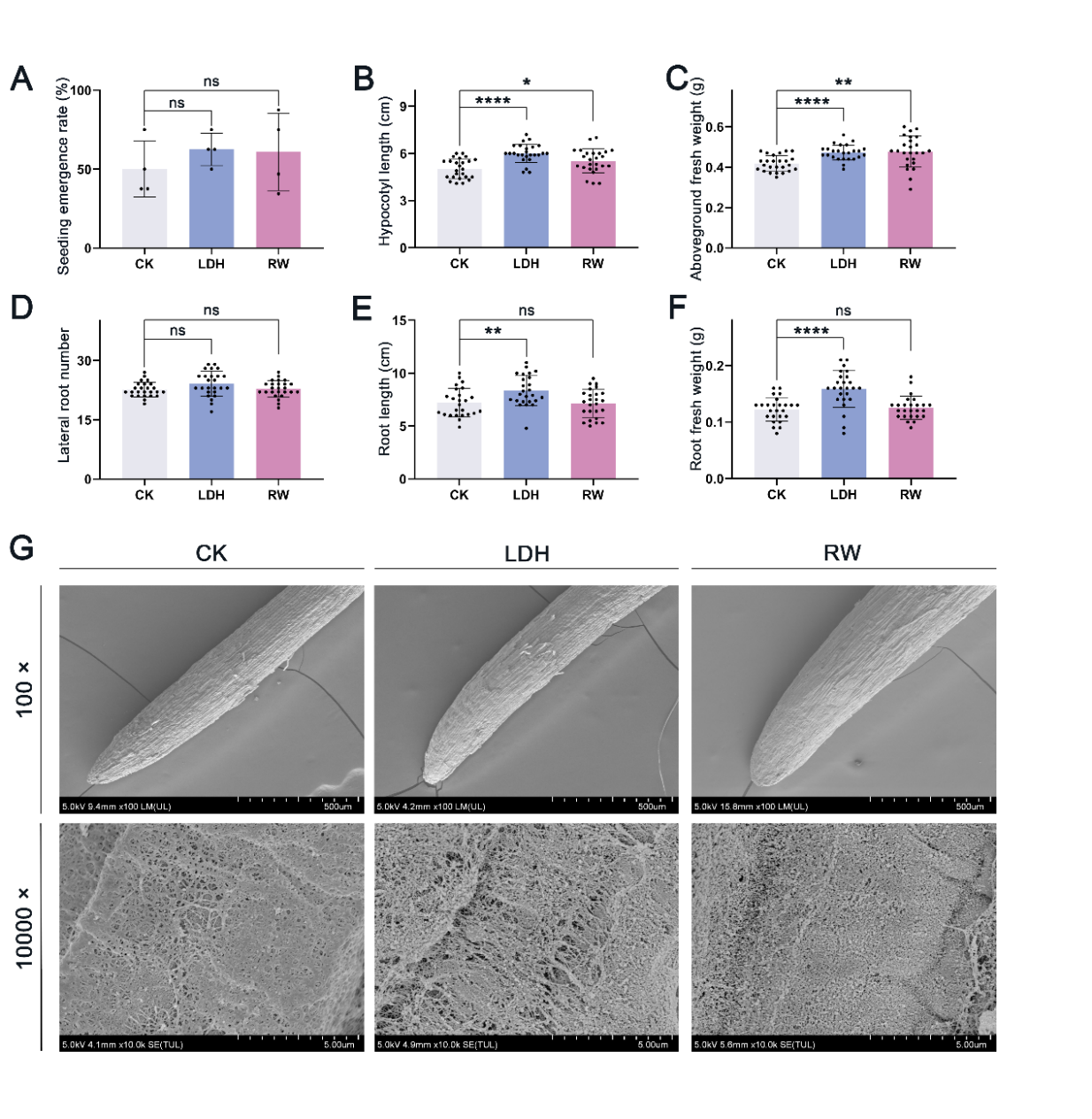
**

**Figure S4** Effects of MgFe-LDHs on seedling growth in room-temperature simulation experiment

The seedling emergence rate (**A**), hypocotyl length (**B**), aboveground fresh weight (C), lateral root number (**E**), root length (**E**) and root fresh weight (**F**) in cucumber roots following foliar exposure to LDH and RW after 10 days in the artificial climate chamber. **p* < 0.05, ***p* < 0.01 and *****p* < 0.0001, Student’s *t* test. The values are presented as the means ± SDs. The abbreviations used are the same as those in Figure S1.

**
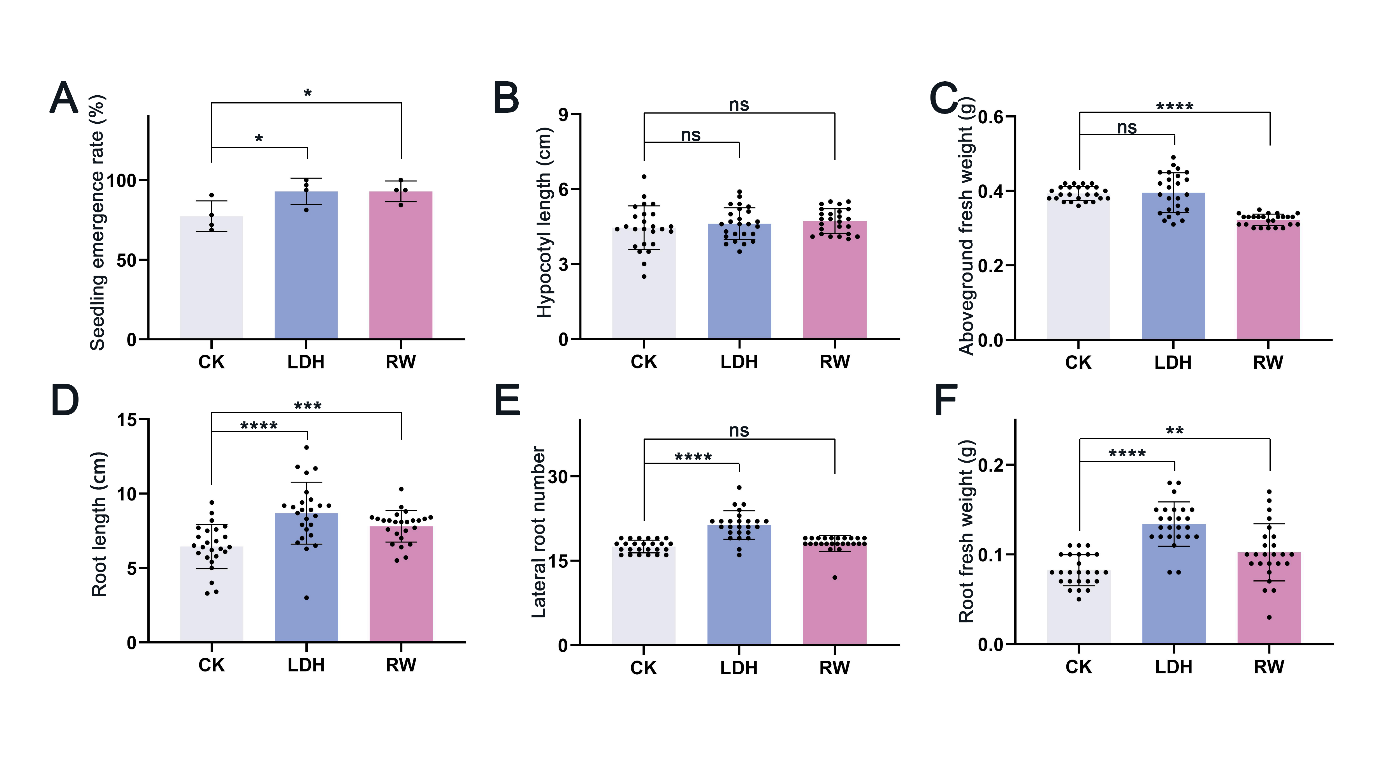
**

**Figure S5** Effects of MgFe-LDHs on seedling growth in low-temperature simulation experiment

The seedling emergence rate (**A**), hypocotyl length (**B**), aboveground fresh weight (C), root length (**D**), lateral root number (**E**), and root fresh weight (**F**) of cucumber roots following foliar exposure to LDH and RW after 10 days in the artificial climate chamber. **p* < 0.05, ***p* < 0.01 and *****p* < 0.0001, Student’s *t* test. The values are presented as the means ± SDs. The abbreviations used are the same as those in Figure S1.


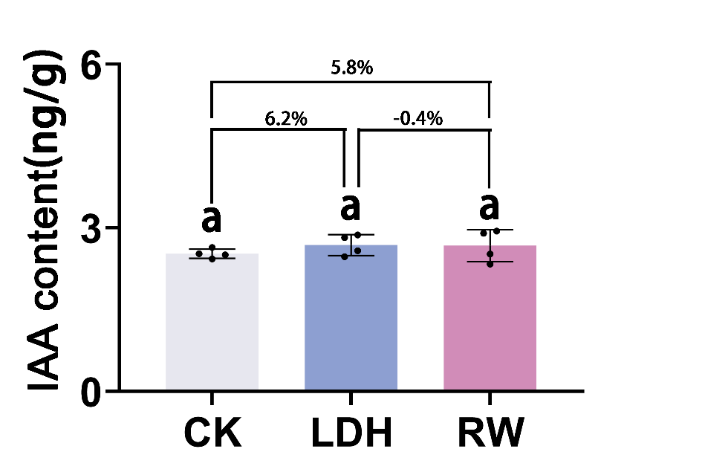


**Figure S6** Mass spectrogram of IAA content

Different lowercase letters indicate significant differences among treatments (*p* < 0.05). The percentages show the magnitude of change among the different treatment groups (LDH/CK, RW/CK, LDH/RW). The abbreviations used are the same as those in Figure S1.


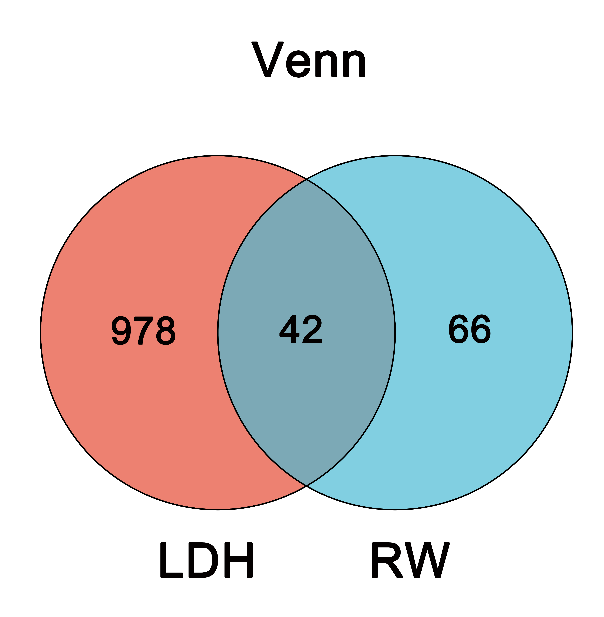


**Figure S7** Venn diagram of the DEGs between different comparison groups

The genes/transcripts in the treatments with a false discovery rate (FDR) less than 0.05 and an absolute fold change ≥ 2 compared to CK were considered differentially expressed genes (DEGs). The abbreviations used are the same as those in Figure S1.


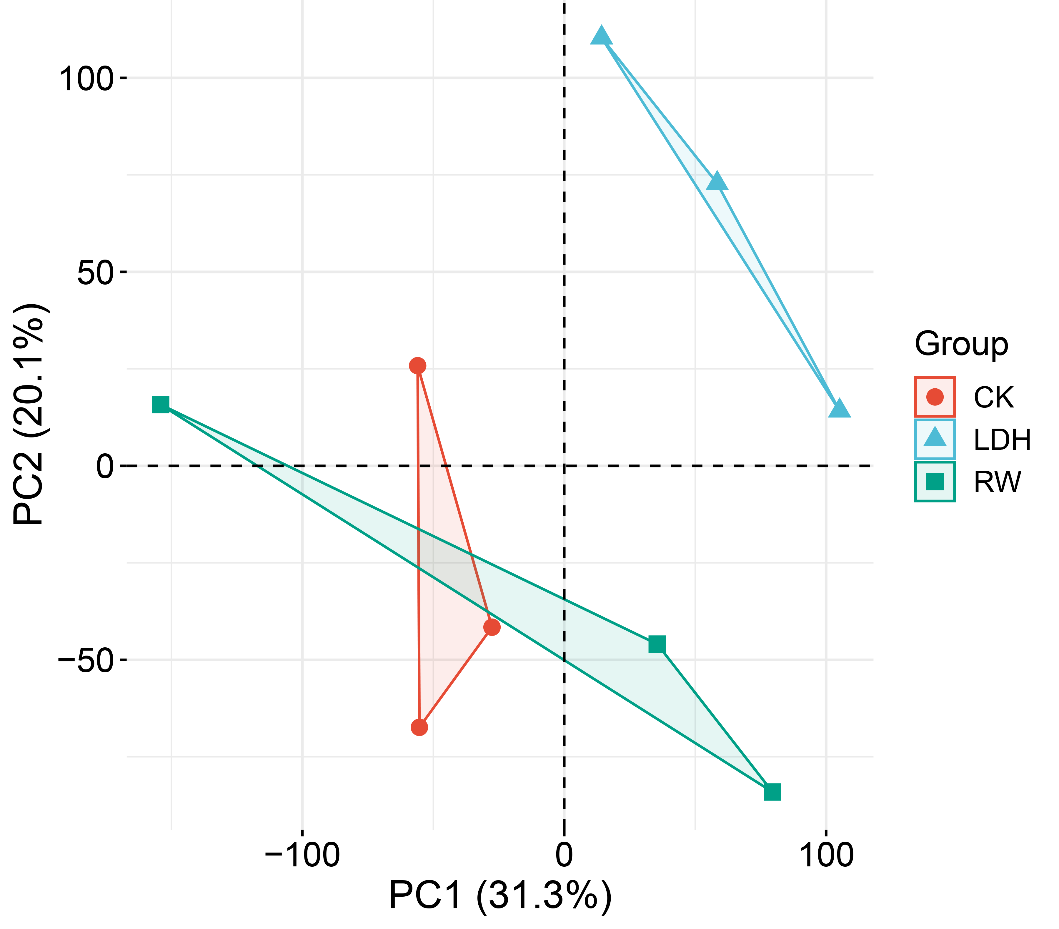


**Figure S8** PCA score plot of the transcriptome of cucumber roots


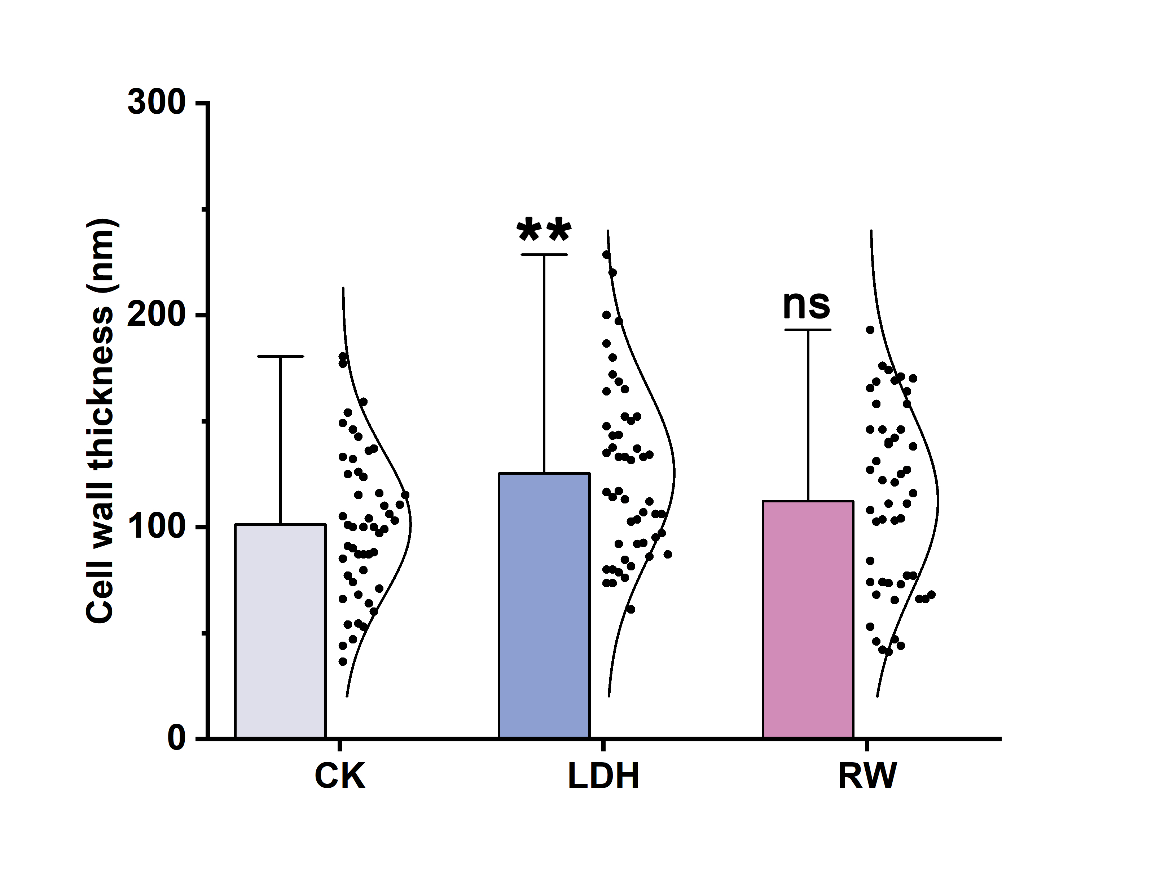


**Figure S9** Cell wall thickness of roots following 10 days of exposure to LDH and RW

Ns, no significance. ***p* < 0.01. Student’s *t* test. The values are presented as the means ± SDs (n=50). The abbreviations used are the same as those in Figure S1.


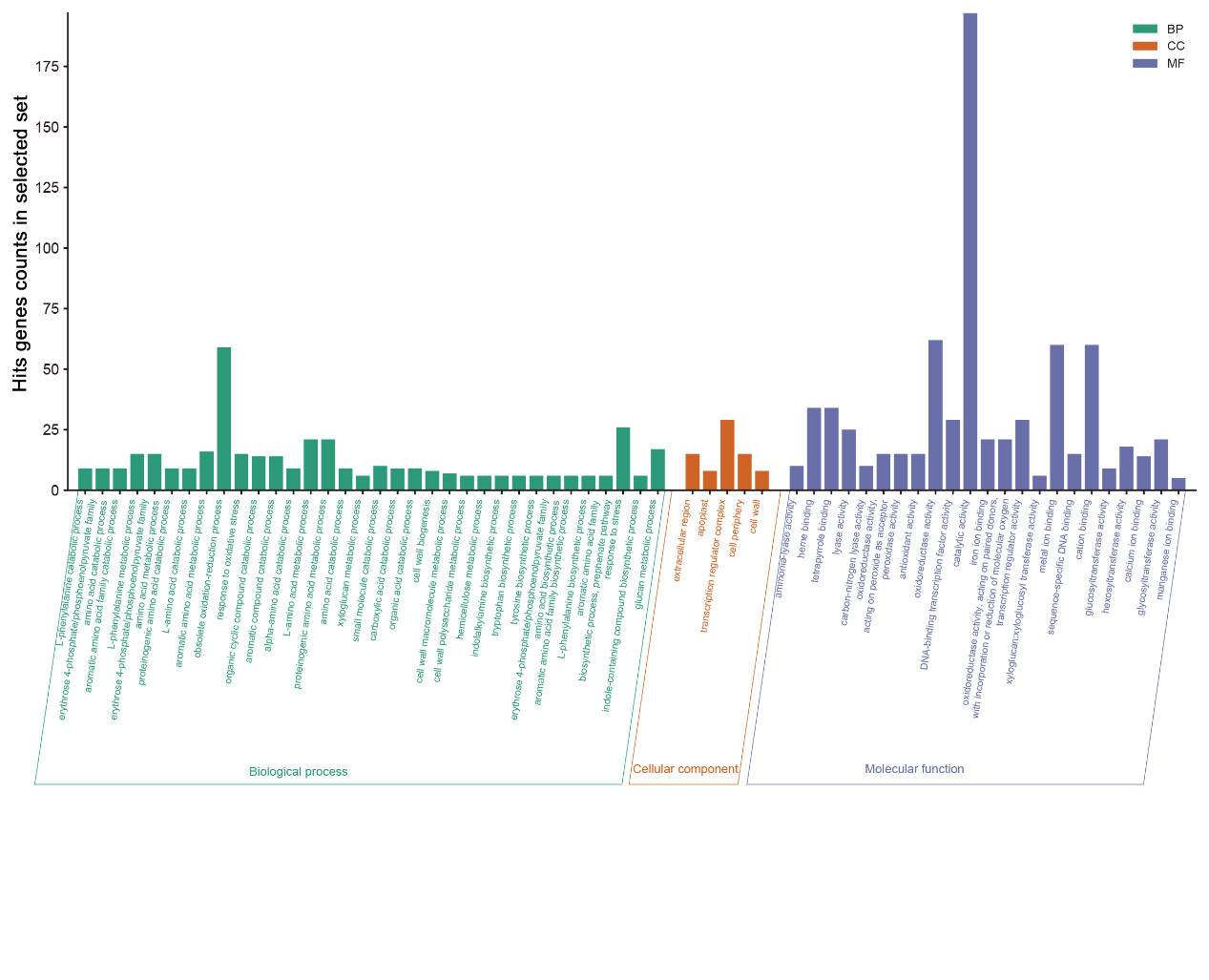


**Figure S10** Enriched GO categories for upregulated DEGs of LDH


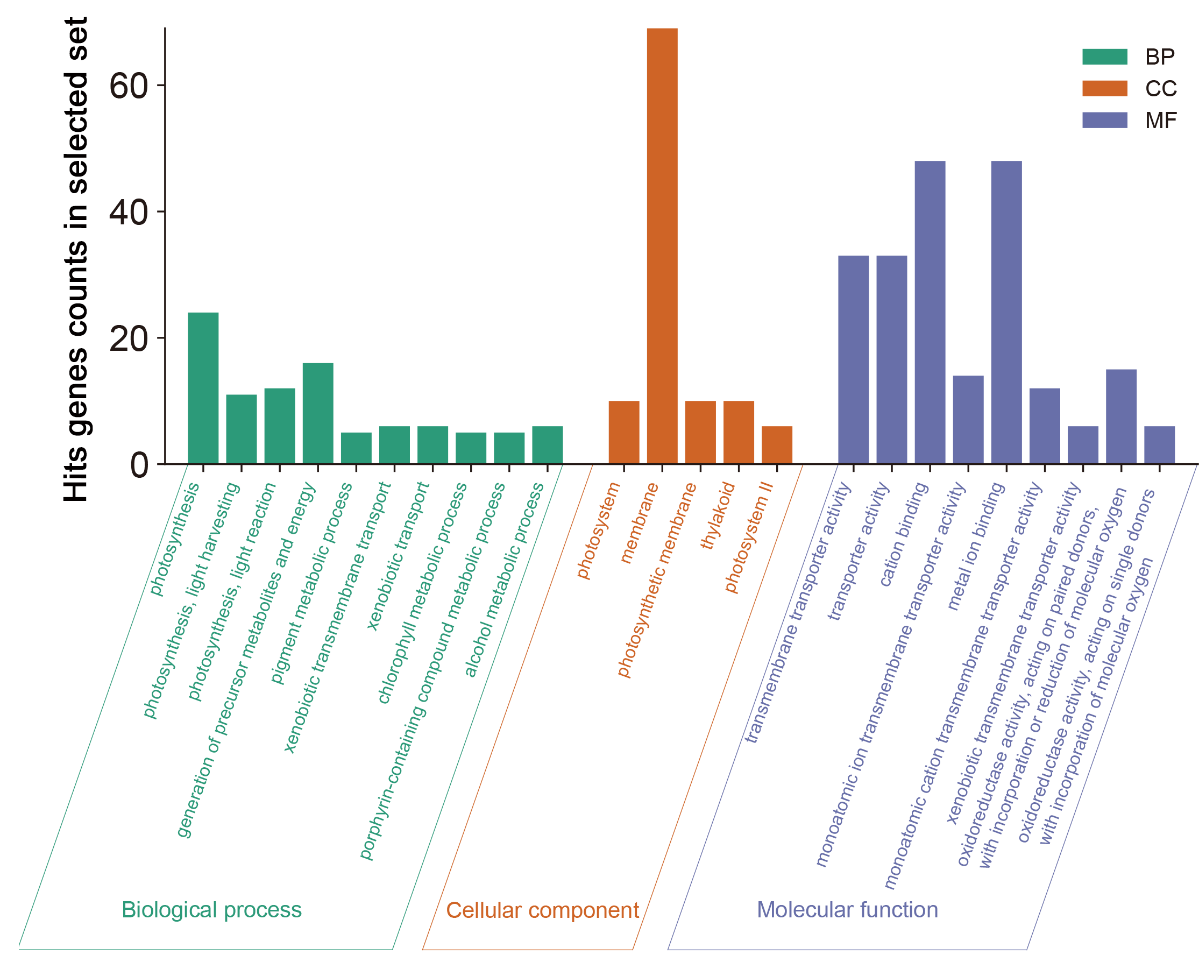


**Figure S11** Enriched GO categories for downregulated DEGs of LDH


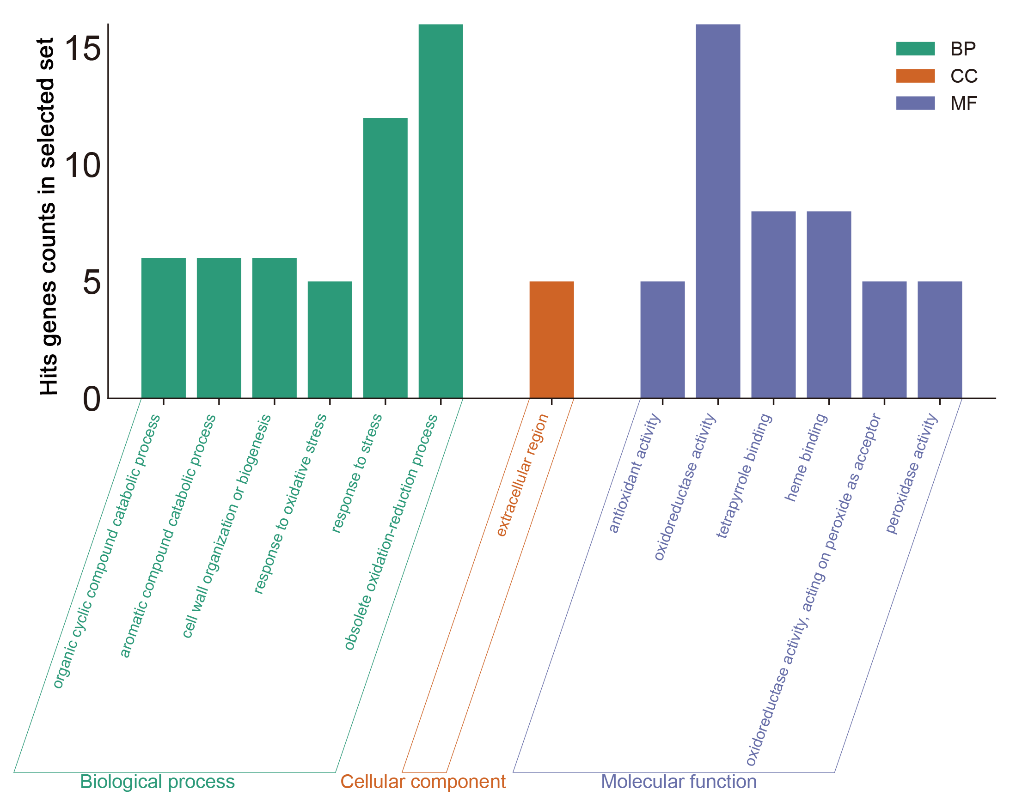


**Figure S12** Enriched GO categories for all DEGs of RW


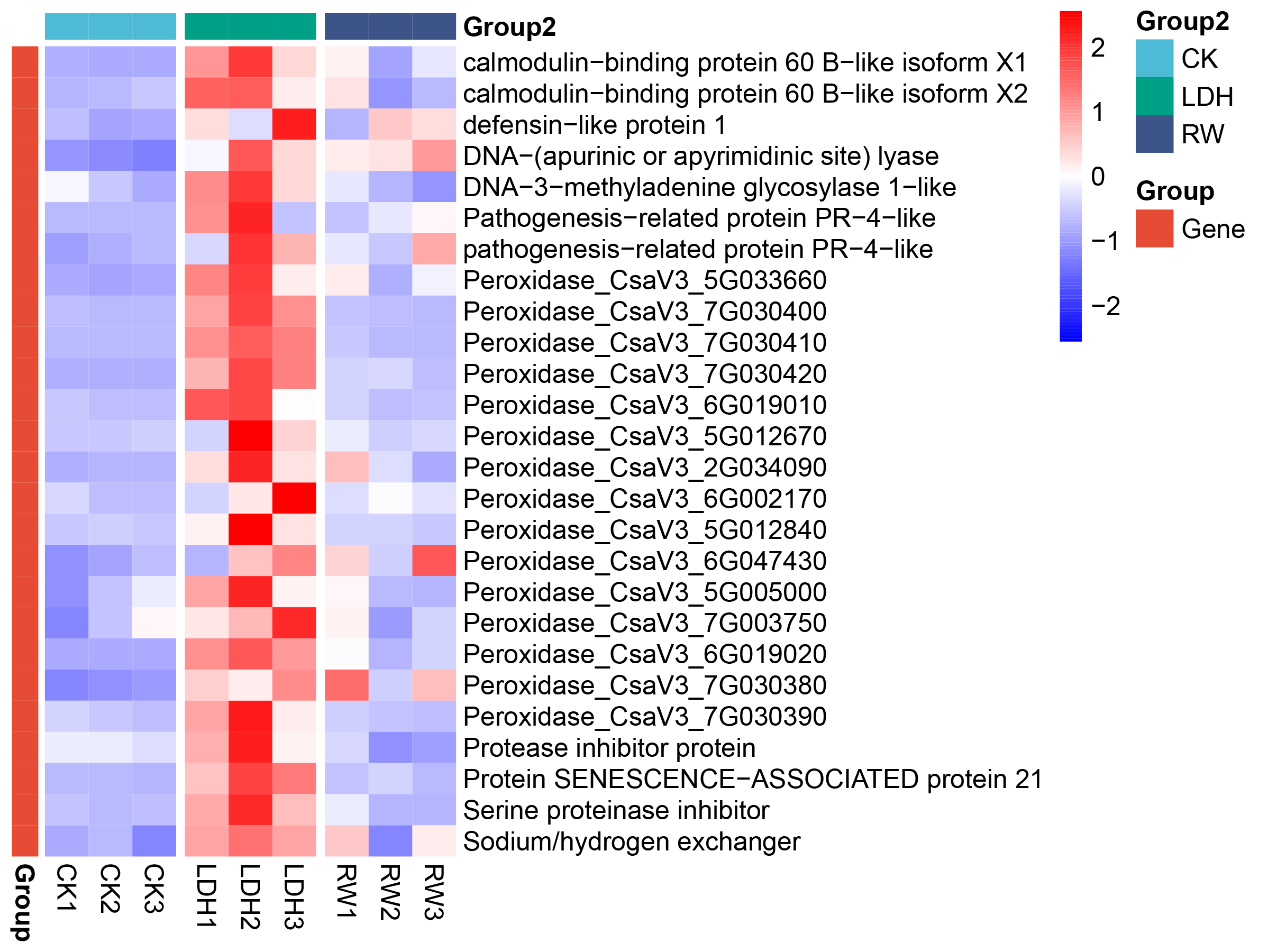


**Figure S13** Stress resistance-related DEGs


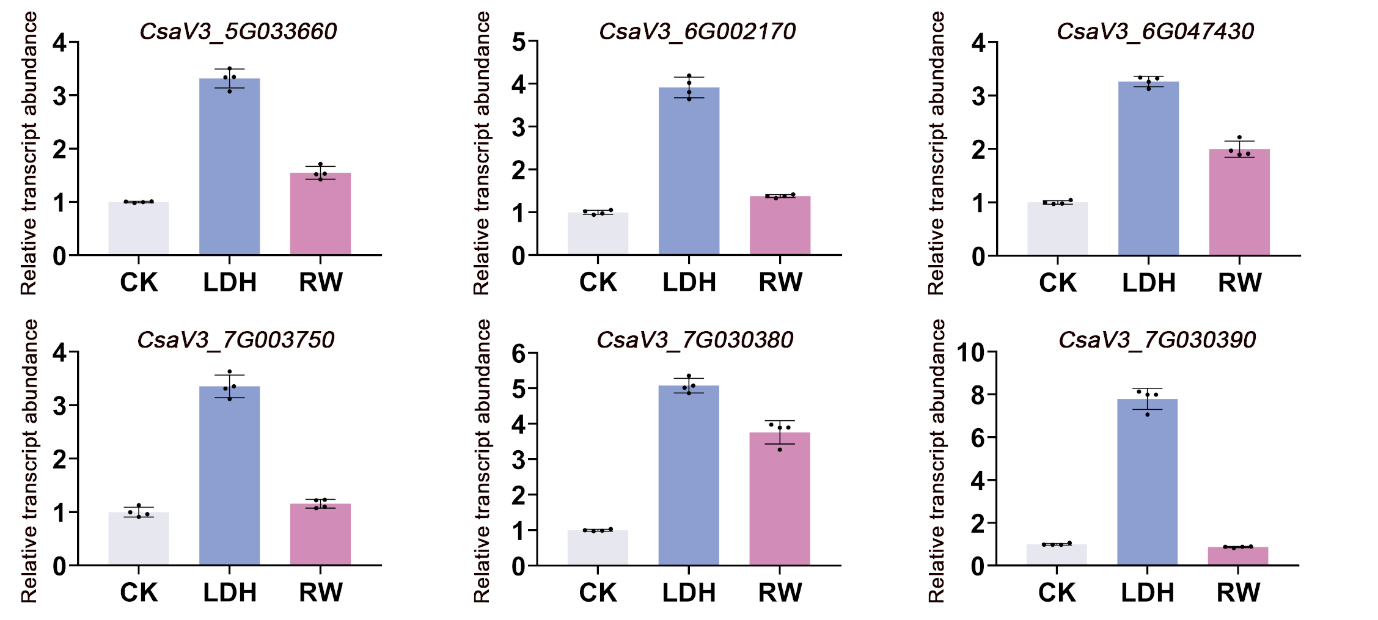


**Figure S14** qRT‒PCR results of DEGs associated with peroxidase


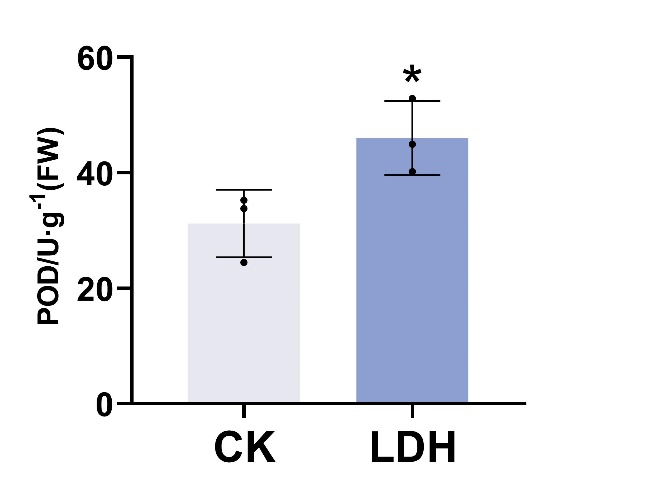


**Figure S15** POD activity

**p* < 0.05. Student’s *t* test. The values are presented as the means ± SDs (n=3). Each treatment group had 3 technical replicates, each containing 3 seedlings. The abbreviations used are the same as those in Figure S1.

## Table

**Table S1** Weather in Beijing

| **Date** | **Lowest/highest temperature** | **Weather conditions** |
| --- | --- | --- |
| 2023-12-8 | -2℃/12℃ | Cloudy to sunny |
| 2023-12-9 | -3℃/8℃ | Cloudy |
| 2023-12-10 | -4℃/0℃ | Overcast to light snow |
| 2023-12-11 | -7℃/-1℃ | Overcast to cloudy |
| 2023-12-12 | -4℃/-2℃ | Cloudy to overcast |
| 2023-12-13 | -5℃/-1℃ | Moderate snow |
| 2023-12-14 | -7℃/-3℃ | Moderate to light snow |
| 2023-12-15 | -13℃/-4℃ | Overcast to cloudy |
| 2023-12-16 | -16℃/-7℃ | Sunny |
| 2023-12-17 | -14℃/-6℃ | Sunny to cloudy |
| 2023-12-18 | -12℃/-4℃ | Overcast to sunny |

**Table S2** ICP‒OES elemental sampling

|  | **Fresh weight (g)** | **Dry weight (g)** | **Water content (%)** |
| --- | --- | --- | --- |
| **CK** | 0.9560 | 0.0467 | 95.11 |
|  | 0.8291 | 0.0383 | 95.37 |
|  | 1.0029 | 0.0492 | 95.09 |
|  | 1.0042 | 0.0491 | 95.11 |
| **LDH** | 1.5921 | 0.0699 | 95.61 |
|  | 1.6085 | 0.0915 | 94.31 |
|  | 1.4222 | 0.0658 | 95.37 |
|  | 1.5077 | 0.0642 | 95.74 |
| **RW** | 1.1403 | 0.0639 | 94.40 |
|  | 1.2007 | 0.0630 | 94.75 |
|  | 1.0804 | 0.0605 | 94.40 |
|  | 1.0145 | 0.0554 | 94.54 |

**Table S3** Primer sequences for qRT‒PCR

| **Gene** | **Forward primer (5'-3')** | **Reverse Primer(5'-3')** |
| --- | --- | --- |
| *CsAct1* | TGGATTCTGGTGATGGTGTGAGT | CTGCTCATAGTCGAGTGCAACATA |
| *CsFAD2.1* | TCTCAAGAAGACAGATTCTGACCAC | AGCCCAGGCTGGATAAAATAAGAG |
| *CsFAD3* | AGAATAAGCCACAGAACTCATCATC | TCACCTGGAGCAAATAAATCACTG |
| *CsaV3_5G033660* | ACCAGAACTTGTCCCAATGTG | GAAACAATCCCAGGACATTCTG |
| *CsaV3_6G002170* | GCATGGGAGCTTCAATTCTTC | AGAACGACAGAATCACGAGC |
| *CsaV3_6G047430* | CAACATGGGATGTTCCTAAAGG | TCGTAGACTTGCTGCAAAGG |
| *CsaV3_7G003750* | ATCCTTGCTTTGATAGCTCGTG | TCGAAGGTCTTGAAGCTTCC |
| *CsaV3_7G030380* | CCACTAACTAGCAACGTTGG | AATCTCGCCTTGTGATCCAG |
| *CsaV3_7G030390* | TTACTTTCCATGCCGAGAGG | TGATGGGCATTTCTCAACACC |

**Table S4** Raw data on the element content (mg/kg)

|  | **CK** | **LDH** | **RW** |  | **CK** | **LDH** | **RW** |
| --- | --- | --- | --- | --- | --- | --- | --- |
| **N** | 75400.0 | 84700.0 | 78700.0 | **K** | 43317.5 | 50784.8 | 43275.2 |
|  | 75300.0 | 84300.0 | 80000.0 |  | 45655.1 | 52454.3 | 37395.4 |
|  | 74400.0 | 83300.0 | 79100.0 |  | 37775.1 | 50088.9 | 53129.1 |
|  | 73800.0 | 82900.0 | 79300.0 |  | 40194.3 | 45758.7 | 44531.2 |
| **P** | 14087.5 | 13467.8 | 15084.0 | **S** | 8353.6 | 9031.7 | 8363.6 |
|  | 12800.7 | 13306.4 | 11864.0 |  | 7474.0 | 9024.9 | 7828.0 |
|  | 13861.8 | 13254.0 | 12767.8 |  | 7495.6 | 8978.6 | 8346.1 |
|  | 11801.4 | 13197.0 | 14294.1 |  | 8413.4 | 8947.7 | 7624.3 |
| **Ca** | 9547.3 | 8048.9 | 9269.3 | **Mg** | 7064.6 | 6355.7 | 7358.4 |
|  | 8691.8 | 9665.2 | 8055.8 |  | 6841.6 | 6614.4 | 7564.3 |
|  | 9199.6 | 10439.0 | 6708.5 |  | 7496.7 | 7196.2 | 6030.2 |
|  | 9359.5 | 8460.3 | 8189.8 |  | 6554.5 | 7194.8 | 5949 |
| **Na** | 1625.3 | 1828.7 | 1388.8 | **Fe** | 155.7 | 285.8 | 86.1 |
|  | 1668.2 | 1671.4 | 1499.9 |  | 152.9 | 277.8 | 138.8 |
|  | 1770.9 | 1861.1 | 1198.9 |  | 179.5 | 210.2 | 139.4 |
|  | 1926.3 | 1820.9 | 1391.8 |  | 170.0 | 188.7 | 190.7 |

**Table S5** Summary of the sequencing reads and read mapping

| **Sample** | **Raw reads** | **Raw bases** | **Clean reads** | **Clean Bases** | **Error%** | **Q20%** | **Q30%** | **GC%** |
| --- | --- | --- | --- | --- | --- | --- | --- | --- |
| CK | 47476828 | 7169001028 | 47126812 | 7071223393 | 0.0122 | 98.61 | 95.65 | 43.58 |
|  | 55121008 | 8323272208 | 54738098 | 8204154274 | 0.0122 | 98.63 | 95.69 | 43.42 |
|  | 56728584 | 8566016184 | 56350200 | 8446129992 | 0.0122 | 98.63 | 95.70 | 43.26 |
| LDH | 62451576 | 9430187976 | 61967230 | 9279887561 | 0.0122 | 98.63 | 95.67 | 43.61 |
|  | 53196748 | 8032708948 | 52820256 | 7913586972 | 0.0122 | 98.62 | 95.66 | 43.60 |
|  | 53525358 | 8082329058 | 53137884 | 7961662120 | 0.0122 | 98.60 | 95.62 | 43.83 |
| RW | 51647324 | 7798745924 | 51271924 | 7680132512 | 0.0122 | 98.63 | 95.72 | 43.71 |
|  | 54232790 | 8189151290 | 53856544 | 8079376462 | 0.0122 | 98.60 | 95.61 | 43.51 |
|  | 55063026 | 8314516926 | 54672692 | 8192202771 | 0.0122 | 98.62 | 95.68 | 43.26 |

Data Filtering and Quality Assessment: Raw RNA sequencing reads were purified using FastP software. After the removal of low-quality reads, adapter sequences, poly(A) tails, and known noncoding RNAs from the raw data, clean reads were obtained. Sample: The designation of the specimen. Raw Reads: Represents the sum of entries in the original sequencing dataset. Raw Bases: Reflects the total volume of unprocessed sequencing information. Clean Reads: The number of entries in the sequencing data was determined after quality filtration. Clean Bases: Indicates the aggregate size of sequencing information following quality control. Error Rate (%): Captures the mean error frequency for bases corresponding to quality-controlled data, typically below 0.1%. Q20 (%) and Q30 (%): These metrics assess the quality of sequencing data after filtration. Q20 and Q30 signify the proportions of bases with sequencing qualities exceeding 99% and 99.9%, respectively, within the total base pool. Generally, Q20 should surpass 85%, and Q30 should exceed 80%. GC Content (%): The percentage of combined G and C bases relative to the total base count in the quality-controlled data.
